# Silver(II)-Mediated Surface Degradation and Passivation of Stainless Steel 316, Graphite, and Titanium in Concentrated Nitric Acid

**DOI:** 10.1021/acsomega.6c08088

**Published:** 2026-10-22

**Authors:** Briana R. Schrage, Jisue Braatz, Megan E. Simms, Daniel E. Felton, Jennifer Neu, Harry Meyer, Kristian G. Myhre

**Affiliations:** † Radioisotope Science and Technology Division, 6146 Oak Ridge National Laboratory, 1 Bethel Valley Rd., Oak Ridge, Tennessee 37830, United States; ‡ Nuclear Nonproliferation Division, 6146 Oak Ridge National Laboratory, 1 Bethel Valley Rd., Oak Ridge, Tennessee 37830, United States; § Chemical Sciences Division, Oak Ridge National Laboratory, 1 Bethel Valley Rd., Oak Ridge, Tennessee 37830, United States

## Abstract

Electrochemically generated Ag­(II) in concentrated nitric acid is a powerful oxidizing medium for mediated electrochemical oxidation, but its practical use requires construction materials that can tolerate highly aggressive redox conditions without excessive degradation or contamination of the process stream. Here, the material-specific responses of stainless steel 316, graphite, and titanium were evaluated in Ag­(II)/8 M HNO_3_ electrolytes generated from 0.01 to 0.5 M AgNO_3_ using an H-cell configuration. Material performance was assessed by mass change measurements, surface profilometry, scanning electron microscopy, Raman spectroscopy, X-ray diffraction, and X-ray photoelectron spectroscopy. SS316 showed concentration-dependent degradation. Graphite exhibited a competing response, with mass gain associated with surface oxidation and Ag incorporation, followed by cracking, crumbling, and particle release under the most aggressive exposure condition. In contrast, titanium showed minimal mass loss and little morphological change, indicating that its TiO_2_ passive layer remained stable in Ag­(II)/HNO_3_. These results show that material compatibility in Ag­(II)-mediated electrochemical oxidation is governed by material-specific surface chemistry and identify titanium as the most robust candidate among the tested materials, while SS316 and graphite require more restricted use depending on mediator concentration and component function.

## Graphical abstract

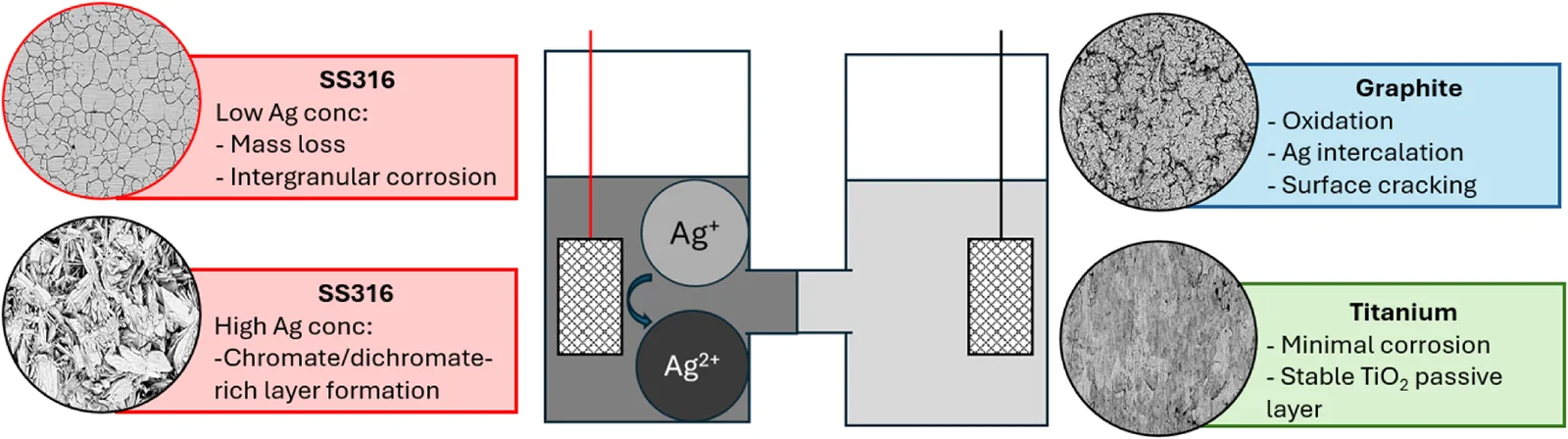

## Introduction

Electrochemically generated high-valent metal ions provide strongly oxidizing acidic media for mediated electrochemical oxidation reactions. In mediated electrochemical oxidation, a redox-active metal ion is oxidized at the anode and subsequently transfers oxidative equivalents to a substrate in solution, after which the reduced mediator can be regenerated electrochemically. This indirect oxidation pathway enables chemical oxidation under comparatively mild operating temperatures and pressures and has been investigated for the treatment of industrial,^1–3^ wastewater streams,^4,5^ hazardous,^6–8^ and radioactive waste streams.^9–12^

Among the metal-ion mediators used for these processes, the Ag­(I)/Ag­(II) redox couple is especially attractive because of the high oxidizing strength of Ag­(II) in nitric acid.^7,12–16^ However, the same redox properties that make Ag­(II) effective for oxidative chemistry also generate a demanding chemical environment for materials exposed to an electrolyte. In concentrated HNO_3_ containing electrochemically generated Ag­(II), construction materials may undergo oxidative dissolution, passive film disruption, secondary surface precipitation, intercalation, or oxide-layer stabilization. These surface reactions can affect cell performance, contaminate process streams, and limit the practical deployment of Ag­(II)-based electrochemical oxidation systems.

Understanding the material-specific surface chemistry in Ag­(II)/HNO_3_ media is therefore essential for selecting components, such as electrodes, electrode supports, cell bodies, liners, flow frames, and ancillary process hardware. Noble or refractory materials such as Ta, Pt, and Nb can provide high corrosion resistance,^17^ but practical electrochemical systems often require more accessible engineering materials. Stainless steel 316, graphite, and titanium represent three chemically distinct material classes that are commonly considered for acidic electrochemical environments. SS316 is an Fe–Cr–Ni alloy with favorable mechanical properties and broad industrial use,^18,19^ but it can undergo localized and intergranular attack in strongly oxidizing nitric acid. Graphite is conductive and chemically useful in electrochemical systems, but its layered structure may permit oxidation, intercalation, and mechanical degradation under highly oxidizing conditions.^20,21^ Titanium, in contrast, is protected by a stable TiO_2_ passive layer that can provide resistance in oxidizing acidic environments.^22,23^

Although Ag­(II)-mediated electrochemical oxidation has been studied for substrate destruction and process chemistry, the concentration-dependent surface response of common engineering materials in electrochemically generated Ag­(II)/HNO_3_ media remains insufficiently understood. In particular, direct comparisons of alloy dissolution, carbon oxidation/intercalation, and passive oxide stability under the same Ag­(II)-generating conditions are limited. Establishing these mechanisms is important not only for material compatibility but also for understanding how highly oxidizing redox mediators modify solid surfaces in concentrated acid.

Here, we compare the surface degradation and passivation behavior of SS316, graphite, and Ti exposed to electrochemically generated Ag­(II) in 8 M HNO_3_. Ag­(II) was generated from AgNO_3_ solutions with initial Ag­(I) concentrations of 0.01–0.5 M, and the exposed materials were characterized by mass-change measurements, scanning electron microscopy, optical surface profilometry, Raman spectroscopy, X-ray diffraction, and X-ray photoelectron spectroscopy. The results reveal three distinct responses: concentration-dependent oxidative attack and Ag–Cr–O surface-layer formation on SS316, oxidation and Ag intercalation in graphite, and retention of Ti surface stability through TiO_2_ passivation. These findings identify the surface chemical pathways that govern material performance in Ag­(II)/HNO_3_ electrolytes and provide a basis for selecting materials for Ag­(II)-mediated electrochemical oxidation systems.

## Experiments

### Materials

AgNO_3_ (99.9%, Thermo Scientific Chemicals) was used to prepare the Ag­(II) stock solutions used in this work. HNO_3_ (trace metal grade, Fisher Chemical) was used without further purification. SS316 and titanium coupons were machined from bulk material at an in-house machine shop and weighed approximately 2.35 g, and titanium grade 2 coupons were machined and weighed approximately 1.42 g. The coupons were 2 × 1 × 0.15 cm thick with a 0.20 cm diameter hole for hanging the coupons. The graphite (Mersen 2114) coupons weighed approximately 0.89 g. The cylindrical coupons were 2.5 cm long with a diameter of 0.5 cm and a 0.60 mm diameter hole for hanging the coupons.

### Electrochemical Setup

The electrochemical oxidation of Ag was carried out in a custom-built H-cell (Figure S1). The compartments accommodated 400 mL each in the anolyte and catholyte chambers and were kept separate by a proton-exchange membrane (Nafion 117). Pt-coated Ti mesh (70 cm^2^) and a Pt-coated Ti rod (30 cm^2^) were used as the anode and cathode, respectively. All experiments were conducted with the H-cell under constant voltage (2.5 V) using a Multicomp Pro DC power supply (0–30 V/0–30 A). The anolyte solution was continuously stirred on a stir/hot plate using a magnetic stir bar at 700 rpm. The catholyte solution was equipped with an air bubbler to regenerate HNO_2_ to HNO_3_.

### Ag­(II) Quantification Titrations

The concentration of Ag­(II) was determined by cerium redox titration. A Mettler Toledo Titrator Excellence T5 equipped with a Pt-ring sensor DMi140-SC electrode was used for all Ce redox titrations. Aliquots of Ag­(II) from experiments were immediately reacted with a 0.1 M stock solution of Ce­(NO_3_)_3_ in 2 M HNO_3_ in a glass titration beaker. The resultant Ce­(IV) was titrated by using a standard ferrous ammonium sulfate solution.

### Corrosion Testing Procedures

The corrosion of SS316, graphite, and Ti was performed as follows. First, solutions of 400 mL of 0.01, 0.05, 0.1, and 0.5 M AgNO_3_ in 8 M HNO_3_ were prepared as the anolyte. The catholyte contained 8 M HNO_3_. Ag­(I) was electrochemically oxidized to Ag­(II) over a span of 60 min under constant voltage (2.5 V), resulting in an average current of 1.2 A. Four weighed and marked coupons were placed on Pt wire hangers in the electrochemical cell. One coupon was removed at each interval (1, 3, 6, and 8 h), rinsed with ultrapure water, and allowed to air-dry overnight. The coupon was reweighed and analyzed by SEM, surface mapping microscopy, XPS, Raman spectroscopy, and XRD. Each experimental set was performed once with a fresh anolyte solution for each set of coupons.

### Surface Morphology and Topography Characterization

Surface morphology and topographical features of the SS316, Ti, and graphite coupons following exposure to electrochemically generated Ag­(II) solutions were characterized using a combination of SEM and optical surface profilometry. The complementary use of these techniques enabled the correlation of microstructural features observed by SEM with height variations measured by profilometry.

SEM was performed using a desktop Phenom scanning electron microscope to examine corrosion features, surface deposits, and morphological changes. Samples were analyzed without conductive coatings to preserve native corrosion products. Imaging was conducted at a magnification of 600× with an accelerating voltage of 15 kV. Backscattered electron detection was employed using the full backscattered detector mode to enhance atomic-number contrast and highlight compositional heterogeneities across the exposed surfaces. The working distance was maintained at approximately 6.315 mm, and the microscope chamber was operated under high-vacuum conditions at a pressure of approximately 0.10 MPa. Multiple regions of each coupon were imaged under identical conditions to enable qualitative comparison between materials and exposure durations.

Surface morphology was also measured using a Keyence optical surface profiler. Images were collected at a 25× zoom ratio, providing a lateral field of view corresponding to a 10 μm scale bar. The surface profiler was used to visualize surface roughening, etching depth, localized material removal, and the development of surface features that are not readily quantifiable by SEM alone. Profilometry images were acquired from a representative region of each sample to complement SEM observations and provide qualitative insight into surface height variations induced by Ag­(II)-mediated corrosion.

The combined SEM and surface profilometry analyses provided a more complete assessment of material degradation by capturing fine-scale morphological features and larger-scale topographical changes resulting from exposure to highly oxidizing MEO conditions.

### Raman Spectroscopy

Raman spectra were acquired using a Renishaw inVia confocal Raman microscope equipped with a 532 nm diode-pumped solid-state laser as the excitation source. The laser was focused onto the sample surface through a microscope objective (e.g., 50×), providing a laser spot size on the order of a few micrometers. The laser power at the sample was kept sufficiently low to avoid thermal damage or laser-induced modification of corrosion products.

The scattered Raman signal was collected in a backscattering geometry, dispersed by a grating spectrograph, and detected using a thermoelectrically cooled CCD detector. Spectra were collected over the range of approximately 100–1500 cm^–1^, which covers the characteristic vibrational modes of metal oxides, chromate species, and nitrate reference compounds.

Each spectrum was obtained with an integration time of 10 s, and multiple accumulations were averaged to improve the signal-to-noise ratio. Prior to analysis, spectra were processed by applying baseline correction to remove the fluorescence background as well as cosmic ray removal where necessary.

Measurements were performed directly on the exposed surfaces of the SS316 coupons after electrochemical testing without any additional surface coatings or conductive layers. Reference spectra of AgNO_3_, Na_2_CrO_4_, K_2_Cr_2_O_7_, Ag_2_CrO_4_, and Ag_2_Cr_2_O_7_ were collected under the same instrumental conditions for comparison and peak assignment.

### X-ray Diffraction

PXRD patterns were collected using a PANalytical Empyrean diffractometer equipped with a Cu anode X-ray tube at 45 kV and 40 mA. The iCore and dCore Soller slits had variable opening widths that were set to automatic mode. The PIXcel3D-Medipix3 detector was used in 1D mode. Data were collected from 3° to 120° with a step size of 0.0263° and a counting time of 1597 s.

GIXRD patterns were collected under fixed incident and divergent slits at 0.0625°, a parallel plate collimator of 0.28°, and an ω of 1°. Data were collected from 5° to 100° with a step size of 0.08° and a counting time of 15.840 s.

### X-ray Photoelectron Spectroscopy

XPS was performed using a Thermo Scientific model Nexsa G2 XPS instrument (Waltham, Massachusetts, USA). The instrument uses monochromatic, microfocused Al K_α_ X-rays (1486.6 eV) with a variable spot size (i.e., 30–400 μm). Samples were attached directly to the XPS sample holder using metal clips. Analyses of the samples were performed with the 400 μm X-ray spot size for maximum signal and to obtain an average surface composition over the largest possible area. The instrument has a hemispherical electron energy analyzer equipped with a 128-channel detector system. The base pressure in the analysis chamber is typically 2 × 10^–9^ mbar or lower. Survey spectra (pass energy = 200 eV) were acquired for qualitative and quantitative analysis, and high-resolution core-level spectra (pass energy = 50 eV) were acquired for detailed chemical state analysis. All spectra were acquired with the charge-neutralization flood gun turned on to maintain a stable analysis condition. The flood gun uses a combination of low-energy electrons and Ar ions for optimum charge compensation. The typical pressure in the analysis chamber with the flood gun operating is 2 × 10^–7^ mbar. Data were collected and processed using the Thermo Scientific Avantage XPS software package (v.6.8.1).

## Results and Discussion

### Ag­(II)-Mediated Mass Changes of SS316, Graphite, and Ti

The material response of SS316, Ti, and graphite coupons was evaluated after exposure to electrochemically generated Ag­(II) in 8 M HNO_3_. Under ideal MEO working conditions, acid concentrations ≥8 M nitric acid are preferred as lower acid concentrations result in lower Ag­(II) generation and promote competing cathode reactions, including hydrogen gas formation.^24^ Acid concentrations of 8 M were chosen for the anolyte since it is an optimum concentration for facilitating Ag­(II) production and stabilizing the Ag­(II) nitrate complex.^13^ The electrochemical cell was held under constant voltage (2.5 V) throughout the duration of the experiments. Ag­(II) was generated in the anolyte by electrochemical oxidation of AgNO_3_ solutions with initial Ag­(I) concentrations ranging from 0.01 to 0.5 M. Before the coupons were immersed in the cell, the concentration of Ag­(II) was checked to confirm that the Ag­(II) concentration reached a steady state, consistent with our previous work.^13^ Overall, during the reaction of Ag­(II) with the coupon surfaces, it was assumed that the Ag­(II) concentration decreased slightly due to its highly oxidizing nature and potential to react with surface atoms on the coupons. Coupons were removed after 1, 3, 6, and 8 h of exposure, rinsed, dried, and weighed to determine the net mass change. Control coupons were exposed to 8 M HNO_3_ under the same time intervals in the absence of AgNO_3_/Ag­(II). The control samples exhibited only small mass changes compared with the Ag­(I)-exposed materials, confirming that the electrochemically generated Ag­(II) species, rather than nitric acid alone, was primarily responsible for the observed degradation behavior.

SS316 displayed a strong dependence on the initial AgNO_3_ concentration. At 0.01, 0.05, and 0.1 M AgNO_3_, the SS316 coupons underwent progressive mass loss with increasing exposure time (Table 1 and Figure 1). After 8 h, the measured mass losses were 31.6, 99.4, and 172 mg for coupons exposed to Ag­(II) generated from 0.01, 0.05, and 0.1 M AgNO_3_, respectively. These values correspond to approximately 1.3, 4.2, and 7.3% loss of the initial SS316 coupon mass. Linear fitting of the mass-loss data over the 1–8 h exposure period gave apparent mass-loss rates of approximately 4.0, 12.5, and 21.7 mg h^–1^ for 0.01, 0.05, and 0.1 M AgNO_3_, respectively, showing that SS316 dissolution increased with increasing Ag­(II) generation.

**Table 1:** Results of Ag­(II) Contact with SS316

SS316	Mass loss (mg)				
Contact time (h)	Controls	0.01 M AgNO_3_	0.05 M AgNO_3_	0.1 M AgNO_3_	0.5 M AgNO_3_
1	4.0	2.9	7.3	18.9	26.7
3	1.5	10.3	27.0	62.0	31.8
6	1.6	19.7	54.3	124.5	43.8
8	2.8	31.6	99.4	172.3	54.3

**Figure 1:**
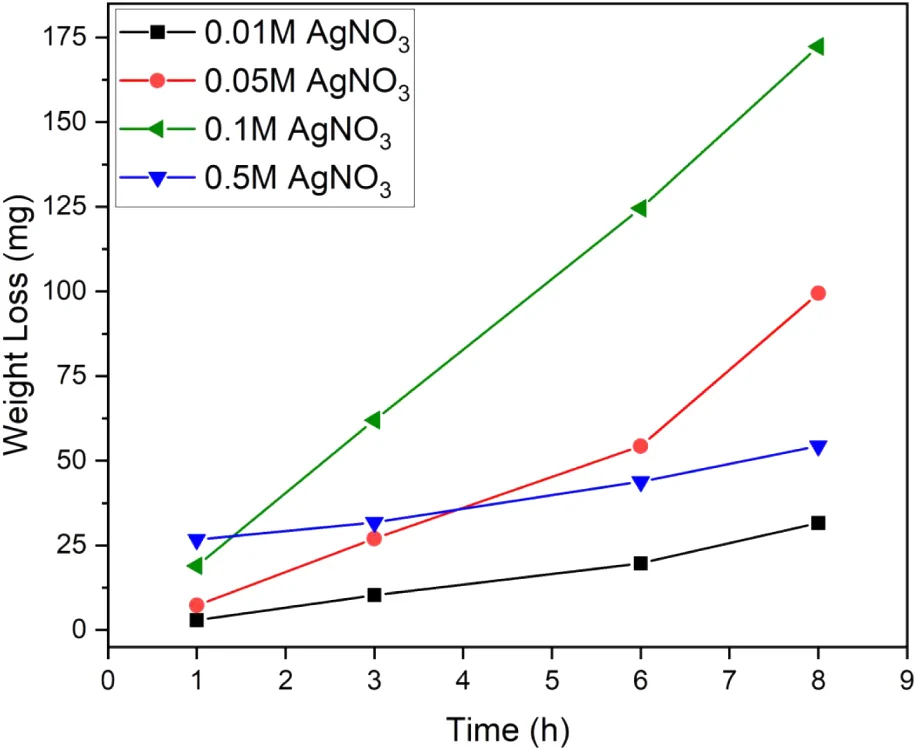
Weight loss of SS316 coupons as a function of AgNO_3_ concentration over time. Conditions: anolyte—initial AgNO_3_ stock = variable AgNO_3_ concentration in 8 M HNO_3_; catholyte-8 M HNO_3_.

The behavior of SS316 at 0.5 M AgNO_3_ differed substantially from the lower-concentration conditions. Although the 0.5 M condition produced an initial mass loss of 26.7 mg after 1 h, the total mass loss after 8 h was only 54.3 mg, which was substantially lower than the 172.3 mg loss observed at 0.1 M AgNO_3_. This nonmonotonic concentration dependence indicates that SS316 degradation is not controlled solely by the total concentration of oxidizing Ag­(II). Instead, at a high AgNO_3_ concentration, a secondary surface reaction becomes important. The SS316 coupons exposed to Ag­(II) generated from 0.5 M AgNO_3_ developed a red crystalline surface layer, suggesting formation of an Ag–Cr–O rich corrosion/deposition product (Figure S6). This layer attenuates further SS316 dissolution during the 8 h exposure period, although longer exposure would be required to determine whether the layer remains protective under practical MEO operating conditions.

Graphite and Ti were evaluated at 0.1 and 0.5 M AgNO_3_, because these conditions produced substantial changes in SS316 and therefore represented more aggressive Ag­(II)-generating environments. In contrast to SS316, graphite generally exhibited mass gain rather than mass loss during the early and intermediate exposure periods (Table 2). At 0.1 M AgNO_3_, the graphite coupons gained 4.3 mg after 1 h and 14.2 mg after 8 h. At 0.5 M AgNO_3_, the graphite gained 10.2, 12.4, and 13.6 mg after 1, 3, and 6 h, respectively. The mass gain is consistent with surface oxidation and Ag incorporation into the graphite structure.^25–29^ However, after 8 h under the 0.5 M AgNO_3_ condition, the graphite coupon showed a net mass loss of 1.3 mg. This transition from mass gain to mass loss was accompanied by visible crumbling and the presence of black particulate material in solution, indicating that oxidation/intercalation-induced structural damage eventually caused mechanical degradation and particle release.

**Table 2:** Results of Ag­(II) Contact with Graphite and Ti

Graphite	Mass gain (mg)		
Contact time (h)	Controls	0.1 M AgNO_3_	0.5 M AgNO_3_
1	0.7	4.3	10.2
3	1.3	6.7	12.4
6	2.6	16.4	13.6
8	1.2	14.2	–1.3 (loss)

Ti	Mass loss (mg)		
Contact time (h)	Controls	0.1 M AgNO_3_	0.5 M AgNO_3_
1	0.1	1.1	0.0
3	0.4	0.6	0.1
6	0.1	0.4	0.4
8	0.1	0.0	0.1

Ti showed the highest stability among the three materials. Across all tested exposure times and AgNO_3_ concentrations, Ti mass changes were minimal and comparable to those of the control samples. At 0.1 m AgNO_3_, the measured Ti mass loss decreased from 1.1 mg after 1 h to 0.0 mg after 8 h. At 0.5 M AgNO_3_, the Ti mass loss remained between 0.0 and 0.4 mg over an 8 h exposure period. These results indicate that Ti resists Ag­(II)-mediated degradation under the tested conditions, most likely because of the persistence of a stable TiO_2_ passive surface layer in the highly oxidizing nitric acid electrolyte.

### Surface Morphology and Topography after Ag­(II) Exposure

Optical surface profilometry and SEM were used to determine how the mass-change behavior correlates with surface morphology. Pristine SS316 and SS316 control samples exposed only to 8 M HNO_3_ retained a polished metallic appearance with only minor surface defects. In contrast, SS316 coupons exposed to Ag­(II) generated from 0.01, 0.05, and 0.1 M AgNO_3_ rapidly developed a dull, etched surface (Figures S3–S5). Surface profiler images showed that corrosion initiated within the first hour and was concentrated along grain boundaries (Figure 2). With increasing exposure time, these grain boundary features became deeper and more pronounced, while the grain interiors remained comparatively less affected. This morphology is characteristic of intergranular corrosion.

**Figure 2:**
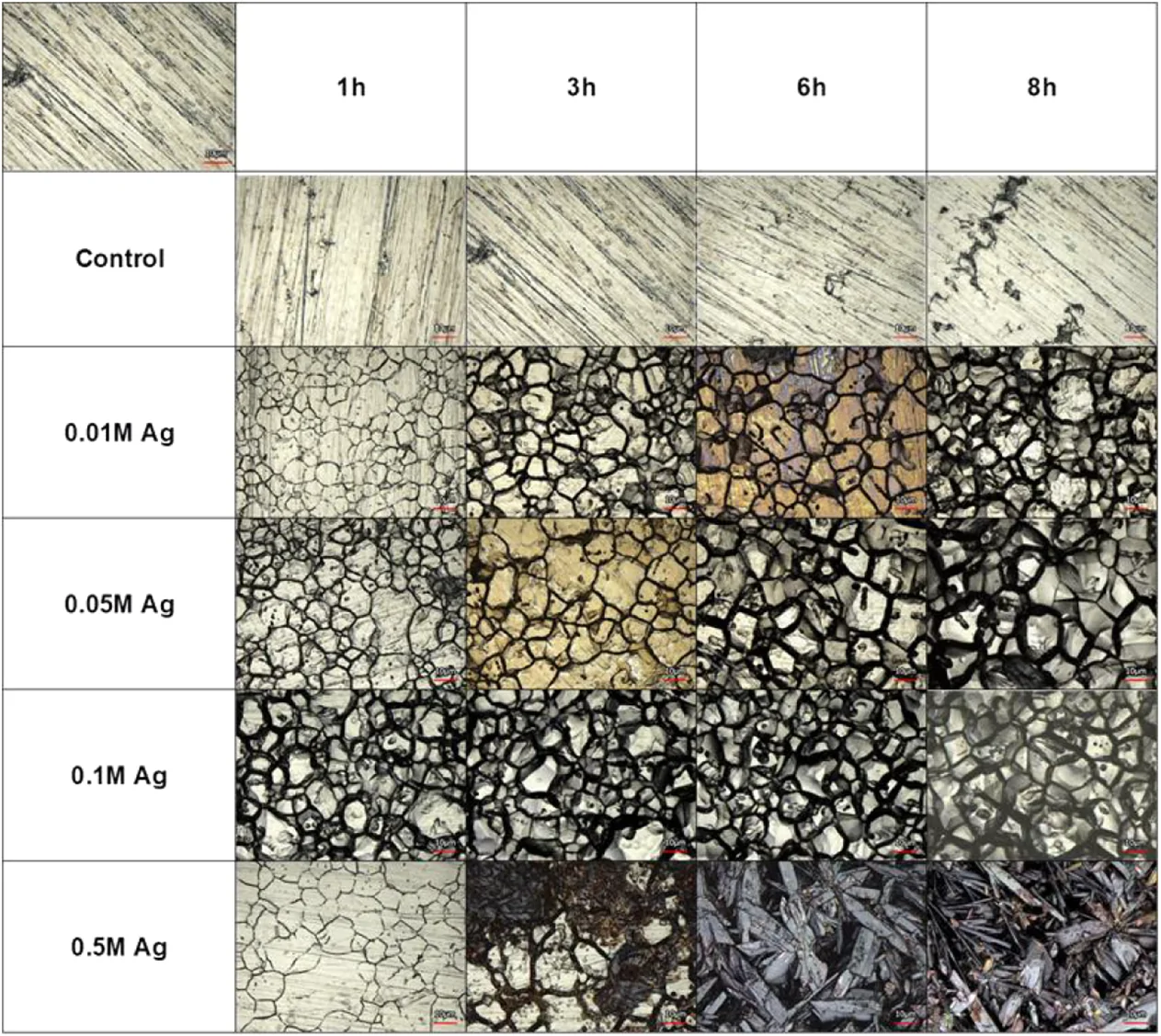
Surface profiler images of SS316 surfaces at 25× magnification. Pristine SS316 is shown in the top corner. Control samples were exposed to 8 M HNO_3_. Samples of SS316 were exposed to Ag­(II) over time. Conditions: initial AgNO_3_ stock = variable AgNO_3_ concentration (0.01, 0.05, 0.1, and 0.5 M) in 8 M HNO_3_, catholyte solution = 8 M HNO_3_, temperature = 25 °C.

The intergranular corrosion observed in SS316 is consistent with the known susceptibility of austenitic stainless steels to grain-boundary attack in a strongly oxidizing nitric acid environment. Although SS316 is generally corrosion-resistant under moderately oxidizing conditions, localized Cr depletion, elemental segregation,^30–32^ or increased defect density near grain boundaries can produce regions that are more susceptible to oxidants that repeatedly destabilize or penetrate the passive oxide film, leading to preferential attack at these chemically and structurally vulnerable grain-boundary regions. Similar intergranular corrosion behavior has been reported for stainless steel exposed to highly oxidizing Ce­(IV)/HNO_3_ decontamination solutions, where the oxidizing mediator accelerates corrosion through anodic polarization of the stainless steel surface.^33,34^

The SS316 surface morphology at 0.5 M AgNO_3_ was distinct from that observed at 0.01 to 0.1 M AgNO_3_. Instead of showing only progressively deepening grain boundary corrosion, the 0.5 M samples developed faceted, plate-like crystalline features across the surface (Figures 2 and 3). These deposits became more extensive with increasing exposure time and produced the red crystalline appearance observed visually. SEM/EDS mapping (Figures S16–S20) showed enrichment of Ag and Cr within these surface features, supporting assignment of the layer as an Ag–Cr–O-rich corrosion/deposition product. The formation of this secondary layer explains the lower net mass loss observed at 0.5 M AgNO_3_ compared with 0.1 M AgNO_3_. Under high Ag­(II)-generating conditions, Cr released from the stainless steel surface can be oxidized to higher-valent chromate/dichromate species,^30^ which then react with Ag-containing species in solution to form sparingly soluble Ag–Cr–O phases on the coupon surface.

**Figure 3:**
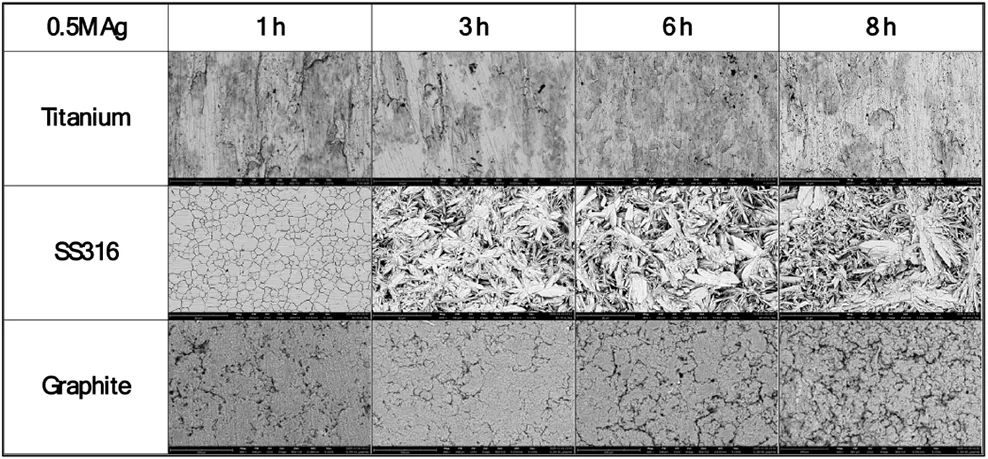
SEM images at 600× magnification for the samples that were exposed to higher Ag­(II) concentrations. Conditions: initial AgNO_3_ stock = variable AgNO_3_ concentration (0.5 M) in 8 M HNO_3_, catholyte solution = 8 M HNO_3_, temperature = 25 °C.

Graphite exhibited a different degradation pathway. Pristine graphite and graphite control samples retained a dark gray, lustrous appearance with preexisting surface heterogeneity. Upon exposure to Ag­(II), the graphite surface became matte black and roughened. SEM and surface profiler images showed that surface cracking and defect formation increased with exposure time, especially under the 0.5 M AgNO_3_ condition (Figures 3 and Figure S28). SEM/EDS (Figures S21–S23) mapping showed increased oxygen content and the presence of Ag on the exposed graphite surfaces. For graphite exposed to Ag­(II) generated from 0.5 M AgNO_3_, the oxygen content increased from approximately 28% after 1 h to 41% after 8 h. These results support a degradation mechanism in which Ag­(II)-containing species become incorporated into or deposited on the layered carbon structure.

The initial mass gain of graphite is therefore attributed to a combination of surface oxidation and Ag incorporation. However, the layered structure of graphite makes it vulnerable to an intercalation-induced mechanical stress. As oxidation and Ag incorporation progress, expansion, cracking, and weakening of the graphite structure occur. At the most aggressive condition, 0.5 M AgNO_3_ for 8 h, the accumulated damage resulted in crumbling and release of graphite particles into the solution, producing a net mass loss. This behavior indicates that graphite may be chemically and mechanically unstable in concentrated Ag­(II)/HNO_3_ electrolytes, despite its common use as a conductive carbon material in electrochemical systems.

Ti exhibited minimal surface modification after Ag­(II) exposure. Pristine Ti, Ti control, and Ti coupons exposed to 0.1 and 0.5 M AgNO_3_ showed similar surface textures, with polishing marks and no obvious evidence of extensive etching, cracking, or deposit formation (Figure 3 and Figures S7–S9, S29). SEM/EDS mapping showed a Ti/O surface composition consistent with the presence of a TiO_2_ passive layer. The lack of significant mass loss and the minimal morphological change indicate that Ti maintains passivity in the Ag­(II)/HNO_3_ environment over the 8 h exposure period. This behavior contrasts sharply with that of SS316 and graphite and identifies Ti as the most chemically stable of the three tested materials.

### Raman Spectroscopy of Ag–Cr–O Surface Deposits on SS316

Because the 0.5 M AgNO_3_ condition produced a visually distinct red crystalline surface layer on SS316, Raman spectroscopy was performed on SS316 coupons that were electrochemically oxidized in a solution of 0.5 M AgNO_3_ corresponding to exposure times of 1, 3, 6, and 8 h (Figure 4). All exposed samples exhibited pronounced Raman features in the approximately 780–900 cm^–1^ region, which intensified with increasing exposure time, particularly between 1 and 6 h, indicating growth of a Raman-active surface layer. This spectral region is characteristic of the Cr–O stretching mode in chromate and dichromate species. The CrO_4_^2–^ symmetric and antisymmetric Cr–O stretching modes match well with reference spectra of Ag_2_CrO_4_ and Na_2_CrO_4_. The same was seen for the Cr_2_O_7_^2–^ system, which depicts symmetric and antisymmetric stretching modes of the Cr–O_3_, matching well with reference spectra of Ag_2_Cr_2_O_7_ and K_2_Cr_2_O_7_.

**Figure 4:**
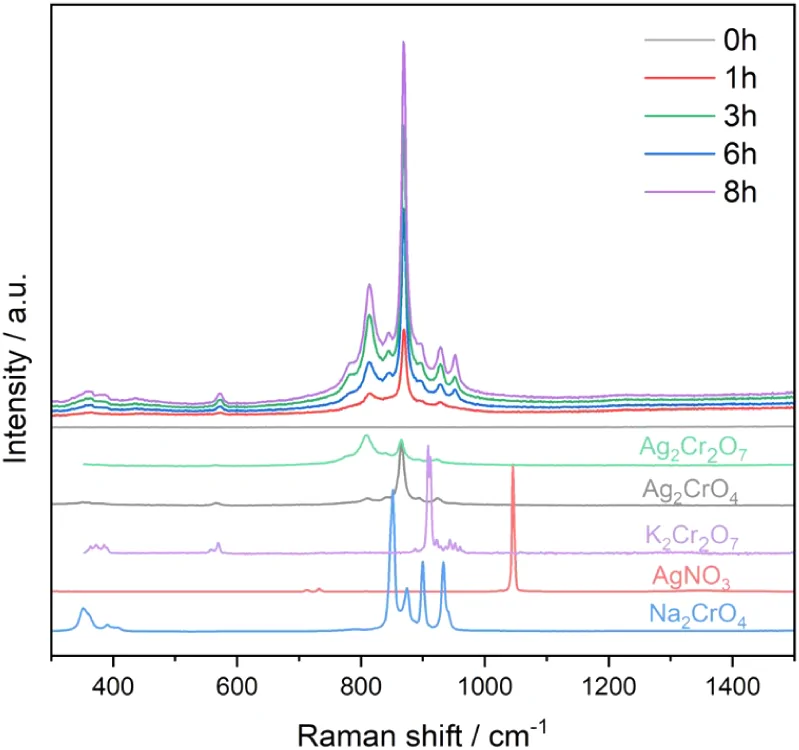
Experimental Raman spectra of samples exposed to electrochemically generated Ag­(II) from 0.5 M AgNO_3_ over time. Samples were compared with reference spectra of AgNO_3_, Na_2_CrO_4_, K_2_Cr_2_O_7_, Ag_2_CrO_4_, and Ag_2_Cr_2_O_7_.

The multipeak structure in this region suggests that the chromate/dichromate species are present in a crystalline or semicrystalline solid environment rather than only as dissolved species. A reproducible band at approximately 571 cm^–1^ was also observed in exposed samples. This feature is consistent with Cr–O–Cr vibrational modes associated with a dichromate-like structure.^35^

The Raman results support the mechanism inferred from mass loss and SEM/EDS analysis. Under highly oxidizing conditions, Cr released from SS316 can be oxidized to high-valent chromate or dichromate species. These species can react with Ag-containing species in the electrolyte to form sparingly soluble Ag–Cr–O deposits on the SS316 surface. The time-dependent growth of the Raman band indicates that this surface layer develops progressively during the exposure.

### X-ray Diffraction of the SS316 Surface Layer

XRD was used to further evaluate the crystalline Ag–Cr–O surface layer formed on SS316 after exposure to the 0.5 M AgNO_3_ condition (Figure 5). The 1 h exposure sample appeared visually heterogeneous, with a shiny central region and a red, matte appearance toward the edges. Conventional powder XRD (PXRD) measurements on this sample showed only reflections attributable to the SS316 substrate, whereas grazing-incidence XRD (GIXRD) measurements (ω = 0.8°) revealed reflections from both Ag_2_CrO_4_ and the SS316 substrate. This result indicates that the surface layer was thin or laterally nonuniform during the early hours.

**Figure 5:**
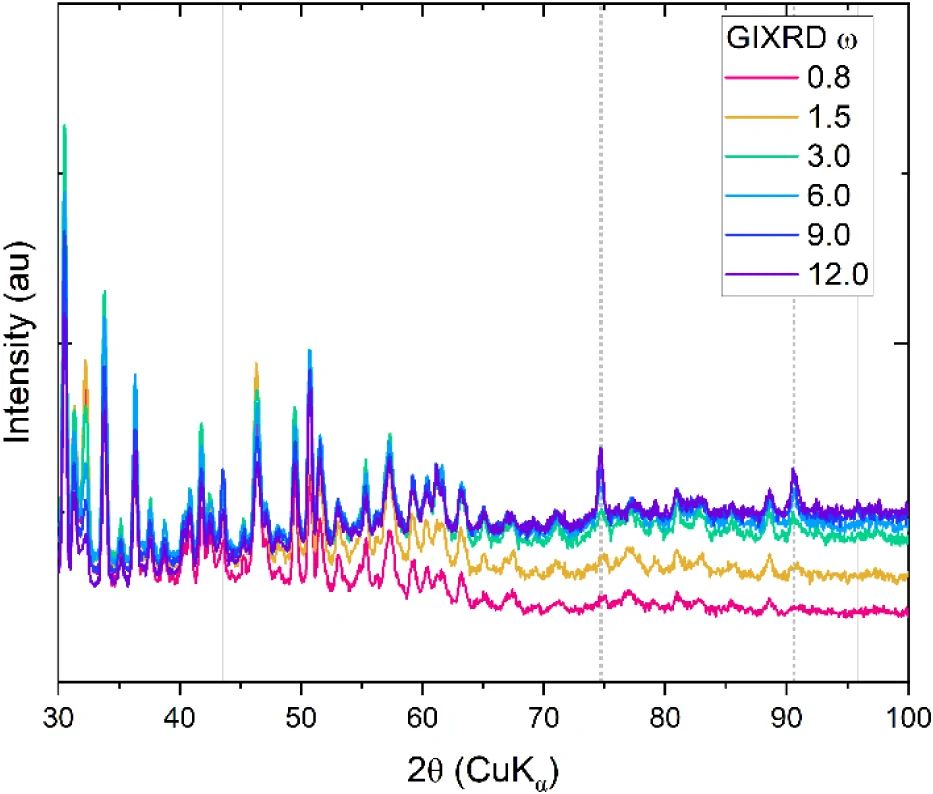
GIXRD patterns obtained using differing ω angles to approximate Ag_2_CrO_4_ layer thickness for the 3 h exposure sample. The Ag­(II) was formed electrochemically from 0.5 M AgNO_3_ solution in 8 M HNO_3_.

A series of GIXRD measurements was performed on the 3 h exposure sample to evaluate the depth scale of the surface layer. At low incident angles, diffraction from the Ag–Cr–O surface layer dominated, while reflections from the SS316 substrate became more evident as the incident angle increased. Distinct SS316 reflections were clearly observed at an incident angle of approximately 6°.

Although some intensity above the background was observed at low ω angles, this is likely attributable to nonuniformity and partial porosity of the Ag_2_CrO_4_ layer, which permitted limited penetration of the incident beam into the substrate. Distinct SS316 reflections became clearly observable at ω ≈ 6°. Using the mass absorption calculator in HighScore for a pure Ag_2_Cr_2_O_7_ surface layer with a calculated density of 4.743 g cm^–3^, an ω angle of 6° corresponds to a probed depth of approximately 2.9 μm for a packing density of 1. The true packing density was expected to be less than unity based on microscopy observations and the residual low-ω substrate intensity; however, the effective penetration depth scales inversely with packing density and can be readily estimated by dividing this value by the assumed fractional density (0 < ρ ≤ 1).

Variable-slit PXRD data from the 8 h exposure sample were imported into GSAS (General Structure Analysis System) for structural refinement. Ag_2_Cr_2_O_7_ was the dominant phase, and all nonsubstrate reflections can be indexed using this structure except for one at 32.2°. We initially attempted to refine the relative phase fractions of potential AgO versus Ag_2_Cr_2_O_7_ using GSAS; however, the low symmetry of Ag_2_CrO_4_ introduced a large number of refinable parameters, and the refinements did not converge as straightforwardly as anticipated. Additionally, SEM suggested the presence of preferred orientation in the Ag_2_CrO_4_ layer, which may further complicate quantitative phase analysis.

Despite these limitations, the presence of AgO was considered unlikely based on direct inspection of the diffraction data. Reflections expected to be unique to AgO (blue hash marks) showed no observable intensity above background, as illustrated in Figure 6. All observed peaks were accounted for by Ag_2_Cr_2_O_7_ (aqua hash marks), except for a weak reflection at approximately 32.2° 2θ that could not be indexed within the Ag_2_Cr_2_O_7_ model. Although this reflection occurs near an expected AgO peak position, the mismatch in 2θ and the absence of other AgO reflections precluded a definitive phase assignment.

**Figure 6:**
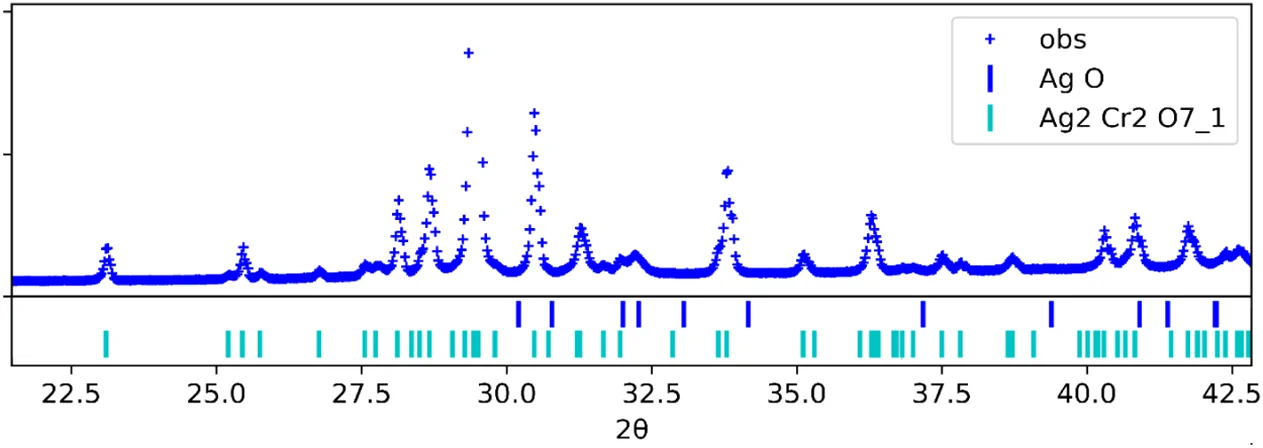
PXRD pattern obtained from the 8 h exposure sample, demonstrating the clear presence of Ag_2_Cr_2_O_7_ and lack of AgO. The Ag­(II) was formed electrochemically from 0.5 M AgNO_3_ solution in 8 M HNO_3_.

Together, the XRD data show that the red crystalline SS316 surface layer formed at 0.5 M AgNO_3_ is dominated by Ag–Cr–O chromate- and dichromate-containing phases. Because Raman and XRD data indicate contributions from both chromate-like and dichromate-like environments, the layer is best described as an Ag–Cr–O chromate/dichromate-rich surface deposit rather than as a single pure phase.

### XPS Analysis of Oxidized SS316 Surfaces

XPS was performed on four samples exposed to Ag­(II) solutions for 1, 3, 6, and 8 h. A control sample that was not exposed to Ag­(II) was also analyzed. Survey spectra confirmed that the presence of Cr, Fe, Ni, Mo, O, and C was observed on all samples as major constituents with the stainless-steel substrate.

Ag was observed on the four exposed samples but not the control, which aligned well with expectations. Regional scans of Ni 2p and Cr 2p showed that the control sample and 1 h exposure sample had Ni metal and relatively small Ni oxide features. The samples exposed for 3 h or more showed only Ni oxide features. The Cr 2p region showed only Cr metal and Cr oxide for all three samples. This region was particularly challenging to resolve because the Ag 3p_3/2_ signal overlaps. The growth of this combined Cr 2p–Ag 3p feature is consistent with the formation of a mixed Ag–Cr–O surface phase. Two distinct peaks were observed in the Ag 3d region for the samples exposed for 3 h or more. The lower-energy peak at 367 eV is likely due to Ag oxide, whereas the other is approximately 1 eV higher in energy and likely related to Ag metal. Deconvolution suggests two chemical states: a lower binding energy component at ∼367.0–367.5 eV (Ag 3d_5/2_), attributed to Ag^+^ species (e.g., Ag–O bonding in oxide or chromate environments), and a higher binding energy component (∼368.0–368.5 eV), consistent with either metallic Ag or weakly coordinated Ag species. The increasing intensity and sharpening of these peaks with time indicate growth and possible crystallization. The O 1s spectra further support this interpretation, showing a dominant lattice oxygen peak (∼529.5–530.5 eV) associated with metal oxides, along with higher binding energy components (∼531–533 eV) attributed to hydroxyl, carbonyl, or adsorbed species. Notably, the relative intensity of lattice oxygen increases with exposure time, consistent with the formation of an oxide-rich surface layer.

Taken together, these results support the Raman, XRD, and SEM-EDS results. Exposure of SS316 to electrochemically generated Ag­(II) in 8 M HNO_3_ causes progressive surface oxidation and the deposition of Ag-containing species. The correlated growth of Ag, Cr, and O features supports the formation of an Ag–Cr–O-rich surface layer. This layer is responsible for the altered SS316 mass-loss behavior observed at 0.5 M AgNO_3_.

### Material-Specific Degradation Pathways in Ag­(II)/HNO_3_

The combined mass change, surface profilometry, Raman, XRD, and XPS results reveal three distinct material responses in electrochemically generated Ag­(II)/HNO_3_ media and are summarized in Table 3. SS316 undergoes concentration-dependent oxidative degradation. At 0.01–0.1 M AgNO_3_, Ag­(II) promotes progressive dissolution of the stainless steel surface, with corrosion concentrated along grain boundaries. This behavior is consistent with intergranular corrosion driven by preferential attack at chemically and structurally vulnerable grain-boundary regions. At 0.5 M AgNO_3_, however, oxidation of Cr released from SS316 and reaction with Ag-containing solution species produce an Ag–Cr–O chromate/dichromate-rich surface layer. This layer changes the dominant surface process from continuous metal dissolution to coupled dissolution, oxidation, and secondary surface precipitation, thereby reducing net mass loss during the 8 h exposure period.

**Table 3:** Summary of Material-Specific Degradation and Passivation Pathways in Electrochemically Generated Ag­(II) /8 M HNO_3_ Media

Material	SS316	Graphite	Ti
Dominant response in Ag(II) solution	Mass loss at 0.01–0.1 M AgNO_3_ solution	Mass gain followed by cracking and particle loss at high Ag(II) exposure	Minimal mass loss and limited surface change
	Lower mass loss with surface deposits at 0.5 M AgNO_3_		
Main surface process	Intergranular corrosion and Ag–Cr–O	Oxidation, Ag incorporation/intercalation, structural degradation	Stable TiO_2_ passivation
	Chromate/dichromate rich layer formation		
Practical implication	Limited compatibility, requires validation for long procedure	Poor choice for structural components in high Ag(II) media	Best-performing candidate among materials tested

Graphite follows an oxidation- and intercalation-driven degradation pathway. Initial exposure to Ag­(II) produces a mass gain because of surface oxidation and incorporation or deposition of Ag-containing species. However, continued oxidation and intercalation damage the layered carbon structure, producing cracks, surface roughening, and eventual particle release. The transition from mass gain to net mass loss for graphite exposed to 0.5 M AgNO_3_ for 8 h indicates that graphite may undergo delayed mechanical failure, even when early mass change measurements appear favorable.

Ti exhibits a passivation-dominated response. The minimal mass loss, unchanged morphology, and persistent Ti/O surface composition indicate that the TiO_2_ passive layer remains stable under the tested Ag­(II)/HNO_3_ conditions. Among the three materials tested, Ti showed the best compatibility with electrochemically generated Ag­(II) in concentrated nitric acid. However, longer-duration experiments and electrochemical impedance or polarization measurements would be valuable for confirming the durability of Ti passivity under practical MEO operation.

## Conclusions

This work demonstrates that candidate materials for Ag­(II)-mediated electrochemical oxidation must be evaluated by both mass change and surface chemical response. In an electrochemically generated Ag­(II)/8 M HNO_3_ solution, titanium showed the best overall compatibility, with minimal mass loss and little morphological change, supporting its use for components that directly contact Ag­(II)-rich electrolyte; SS316 exhibited condition-dependent performance. It is mechanically attractive, but its measurable degradation under low-to-moderate AgNO_3_ concentrations and surface-product formation at high AgNO_3_ concentrations indicate that its use should be limited to carefully selected operating windows. Graphite showed early mass gain but poor structural reliability under aggressive exposure, making it unsuitable for high Ag­(II) concentration where cracking, crumbling, or particulate release cannot be tolerated.

The results establish the following practical compatibility order for the tested materials: Ti > SS316 under selected conditions > graphite under prolonged high Ag­(II) concentration. More broadly, the study shows that compatibility in Ag­(II)/HNO_3_ media is controlled not simply by the corrosion rate but by whether the material dissolves, forms surface deposits, undergoes structural disruption, or maintains passivation. These findings provide a basis for selecting wetted materials for the Ag­(II)-based MEO system and motivate longer-duration, flowing-electrolyte, and cyclic-operation studies to validate performance under practical process conditions.

## Supplementary Material



## Notes

Details of the custom H-cell, coupon images, dissolution rates, surface elemental composition of coupons, surface profiler images, XPS spectra, and the uncertainties and assumptions in GIXRD depth analysis (PDF)
