# Supplementary material for: Silver(II)-Mediated Surface Degradation and Passivation of Stainless Steel 316, Graphite, and Titanium in Concentrated Nitric Acid
Source: ACS Omega. 2026 Oct 22;11(44):68824–32. doi: 10.1021/acsomega.6c08088 (PMC13756720; doi:10.1021/acsomega.6c08088)
Supplement: Supplementary file 1 [file ao6c08088_si_001.pdf]

Silver(II)-Mediated Surface Degradation and Passivation of Stainless Steel 316, Graphite and Titanium in Concentrated Nitric Acid

Briana R. Schrage,[+ a] Jisue Braatz,[+a] Megan E. Simms,[a] Daniel E. Felton,[b] Jennifer Neu,[b] Harry Meyer III[c], and Kristian G. Myhre[a]*

AUTHOR ADDRESS

[a]Radioisotope Science and Technology Division, Oak Ridge National Laboratory, 1 Bethel Valley Rd., Oak Ridge, Tennessee 37831, United States.

[b]Nuclear Nonproliferation Division, Oak Ridge National Laboratory, 1 Bethel Valley Rd., Oak Ridge, Tennessee 37831, United States.

[c]Chemical Sciences Division, Oak Ridge National Laboratory, 1 Bethel Valley Rd., Oak Ridge, Tennessee 37831, United States.

[+] These authors contributed equally to this work.

Supplementary Information

Table of Contents

Figure S1. Setup of the custom-built gas H Cell. Anode = Pt coated Ti mesh, cathode = Pt coated Ti rod, Nafion 117..........**S5**

Figure S2. Picture of the pristine 316 stainless steel and control coupons in nitric acid. Control samples were pulled at 1, 3, 6, and 8 hours. Conditions: 8M HNO_3..........**S6**

Figure S3. Picture of the pristine 316 stainless steel and coupons reacted with Ag(II). Samples were pulled at 1, 3, 6, and 8 hours. Conditions: initial $AgNO_3$ stock = 0.01M $AgNO_3$ concentration in 8M HNO_3, catholyte solution = 8M HNO_3..........**S6**

Figure S4. Picture of the pristine 316 stainless steel and coupons reacted with Ag(II). Samples were pulled at 1, 3, 6, and 8 hours. Conditions: initial $AgNO_3$ stock = 0.05M $AgNO_3$ concentration in 8M HNO_3, catholyte solution = 8M HNO_3..........**S7**

Figure S5. Picture of the pristine 316 stainless steel and coupons reacted with Ag(II). Samples were pulled at 1, 3, 6, and 8 hours. Conditions: initial $AgNO_3$ stock = 0.1M $AgNO_3$ concentration in 8M HNO_3, catholyte solution = 8M HNO_3..........**S7**

Figure S6. Picture of the pristine 316 stainless steel and coupons reacted with Ag(II). Samples were pulled at 1, 3, 6, and 8 hours. Conditions: initial $AgNO_3$ stock = 0.5M $AgNO_3$ concentration in 8M HNO_3, catholyte solution = 8M HNO_3..........**S8**

Figure S7. Picture of the pristine titanium and control coupons in nitric acid. Control samples were pulled at 1, 3, 6, and 8 hours. Conditions: 8M HNO_3. ....**S8**

Figure S8. Picture of the pristine titanium and coupons reacted with Ag(II). Samples were pulled at 1, 3, 6, and 8 hours. Conditions: initial $AgNO_3$ stock = 0.1M $AgNO_3$ concentration in 8M HNO_3, catholyte solution = 8M HNO_3. ....**S9**

Figure S9. Picture of the pristine titanium and coupons reacted with Ag(II). Samples were pulled at 1, 3, 6, and 8 hours. Conditions: initial $AgNO_3$ stock = 0.5M $AgNO_3$ concentration in 8M HNO_3, catholyte solution = 8M HNO_3. ....**S9**

Figure S10. Picture of the pristine graphite and control coupons in nitric acid. Control samples were pulled at 1, 3, 6, and 8 hours. Conditions: 8M HNO_3. ....**S10**

Figure S11. Picture of the pristine graphite and coupons reacted with Ag(II). Samples were pulled at 1, 3, 6, and 8 hours. Conditions: initial $AgNO_3$ stock = 0.1M $AgNO_3$ concentration in 8M HNO_3, catholyte solution = 8M HNO_3. ....**S10**

Figure S12. Picture of the pristine graphite and coupons reacted with Ag(II). Samples were pulled at 1, 3, 6, and 8 hours. Conditions: initial $AgNO_3$ stock = 0.5M $AgNO_3$ concentration in 8M HNO_3, catholyte solution = 8M HNO_3. ....**S11**

Figure S13. Dissolution rate of 316 stainless steel reacted with Ag(II). Samples were pulled at 1, 3, 6, and 8 hours. Conditions: initial $AgNO_3$ stock = 0.01M $AgNO_3$ concentration in 8M HNO_3, catholyte solution = 8M HNO_3. ....**S11**

Figure S14. Dissolution rate of 316 stainless steel reacted with Ag(II). Samples were pulled at 1, 3, 6, and 8 hours. Conditions: initial $AgNO_3$ stock = 0.05M $AgNO_3$ concentration in 8M HNO_3, catholyte solution = 8M HNO_3. ....**S12**

Figure S15. Dissolution rate of 316 stainless steel reacted with Ag(II). Samples were pulled at 1, 3, 6, and 8 hours. Conditions: initial $AgNO_3$ stock = 0.1M $AgNO_3$ concentration in 8M HNO_3, catholyte solution = 8M HNO_3. ....**S12**

Figure S16. Surface elemental composition of pristine 316 stainless steel and coupons in nitric acid. Control samples were pulled at 1, 3, 6, and 8 hours and determined by SEM/EDX analysis. Conditions: 8M HNO_3. ....**S13**

Figure S17. Surface elemental composition of pristine 316 stainless steel and coupons reacted with Ag(II). Samples were pulled at 1, 3, 6, and 8 hours and determined by SEM/EDX analysis. Conditions: initial $AgNO_3$ stock = 0.01M $AgNO_3$ concentration in 8M HNO_3 ....**S14**

Figure S18. Surface elemental composition of pristine 316 stainless steel and coupons reacted with Ag(II). Samples were pulled at 1, 3, 6, and 8 hours and determined by SEM/EDX analysis. Conditions: initial $AgNO_3$ stock = 0.05M $AgNO_3$ concentration in 8M HNO_3 ....**S15**

Figure S19. Surface elemental composition of pristine 316 stainless steel and coupons reacted with Ag(II). Samples were pulled at 1, 3, 6, and 8 hours and determined by SEM/EDX analysis. Conditions: initial $AgNO_3$ stock = 0.1M $AgNO_3$ concentration in 8M HNO_3 ....**S16**

Figure S20. Surface elemental composition of pristine 316 stainless steel and coupons reacted with Ag(II). Samples were pulled at 1, 3, 6, and 8 hours and determined by SEM/EDX analysis. Conditions: initial $AgNO_3$ stock = 0.5M $AgNO_3$ concentration in 8M HNO_3 ....**S17**

Figure S21. Surface elemental composition of pristine graphite and graphite coupons in nitric acid. Control samples were pulled at 1, 3, 6, and 8 hours and determined by SEM/EDX analysis. Conditions: 8M HNO_3. ....**S18**

Figure S22. Surface elemental composition of pristine graphite and graphite coupons reacted with Ag(II). Samples were pulled at 1, 3, 6, and 8 hours and determined by SEM/EDX analysis. Conditions: initial AgNO3 stock = 0.1M $AgNO_3$ concentration in 8M HNO3, catholyte solution = 8M HNO_3. ....**S19**

Figure S23. Surface elemental composition of pristine graphite and graphite coupons reacted with Ag(II). Samples were pulled at 1, 3, 6, and 8 hours and determined by SEM/EDX analysis. Conditions: initial AgNO3 stock = 0.5M $AgNO_3$ concentration in 8M HNO3, catholyte solution = 8M HNO_3. ....**S20**

Figure S24. Surface elemental composition of pristine titanium and titanium coupons in nitric acid. Control samples were pulled at 1, 3, 6, and 8 hours and determined by SEM/EDX analysis. Conditions: 8M HNO_3. ....**S21**

Figure S25. Surface elemental composition of pristine titanium and titanium coupons reacted with Ag(II). Samples were pulled at 1, 3, 6, and 8 hours and determined by SEM/EDX analysis. Conditions: initial $AgNO_3$ stock = 0.1M $AgNO_3$ concentration in 8M HNO_3, catholyte solution = 8M HNO_3. ....**S22**

Figure S26. Surface elemental composition of pristine titanium and titanium coupons reacted with Ag(II). Samples were pulled at 1, 3, 6, and 8 hours and determined by SEM/EDX analysis. Conditions: initial $AgNO_3$ stock = 0.5M $AgNO_3$ concentration in 8M HNO_3, catholyte solution = 8M HNO_3. ....**S23**

Figure S27. Surface profiler images of SS316 surfaces at 25× magnification. **S24**

Figure S28. Surface profiler images of graphite surfaces at 25× magnification. **S25**

Figure S29. Surface profiler images of titanium surfaces at 25× magnification. **S26**

Figure S30. XPS survey spectra of SS316 samples before and after reacted with Ag(II). Samples were pulled at 1, 3, 6, and 8 hours. Conditions: initial $AgNO_3$ stock = 0.5M $AgNO_3$ concentration in 8M HNO_3, catholyte solution = 8M HNO_3**.** **S27**

Figure S31. XPS spectra of (Top left) Cr 2p/Ag 3p, (top right) O 1s, (bottom left) Ag 3d, and (bottom right) Ni 2p regions for SS316 samples before and after reacted with Ag(II). Samples were pulled at 1, 3, 6, and 8 hours. **S28**

Uncertainty and Assumptions in GIXRD Depth Analysis **S29**

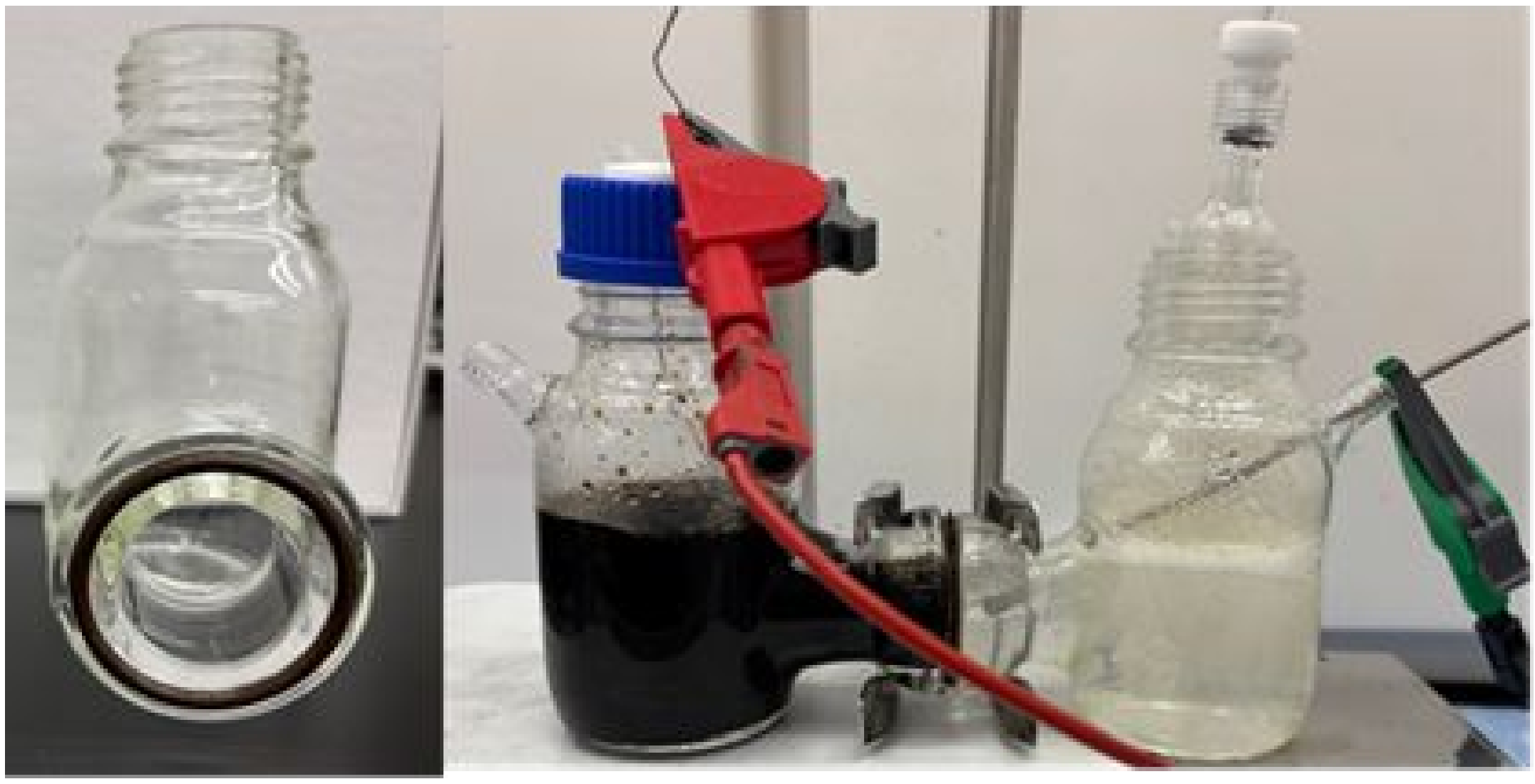

Figure S1. Setup of the custom-built gas H Cell. Anode = Pt coated Ti mesh, cathode = Pt coated Ti rod, Nafion 117.

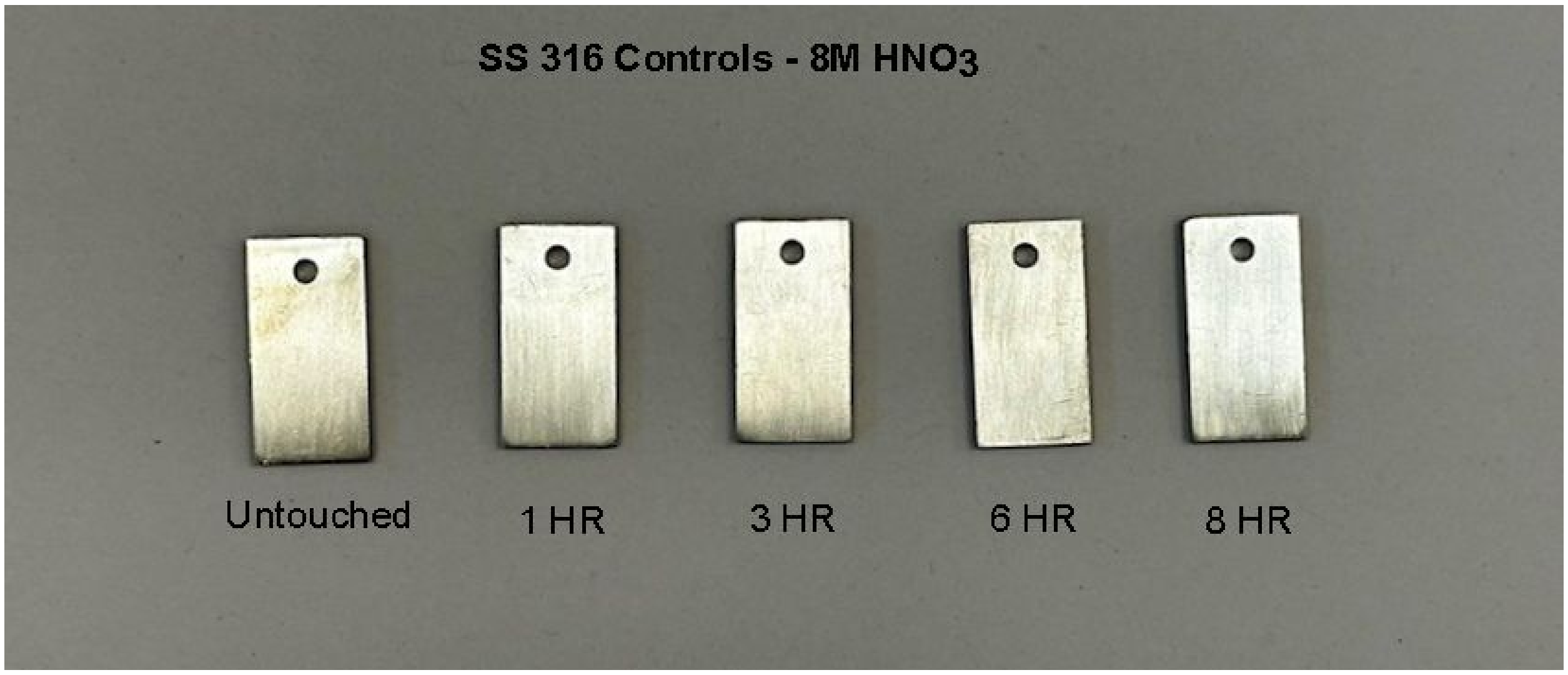

Figure S2. Picture of the pristine 316 stainless steel and control coupons in nitric acid. Control samples were pulled at 1, 3, 6, and 8 hours. Conditions: 8M HNO_3.

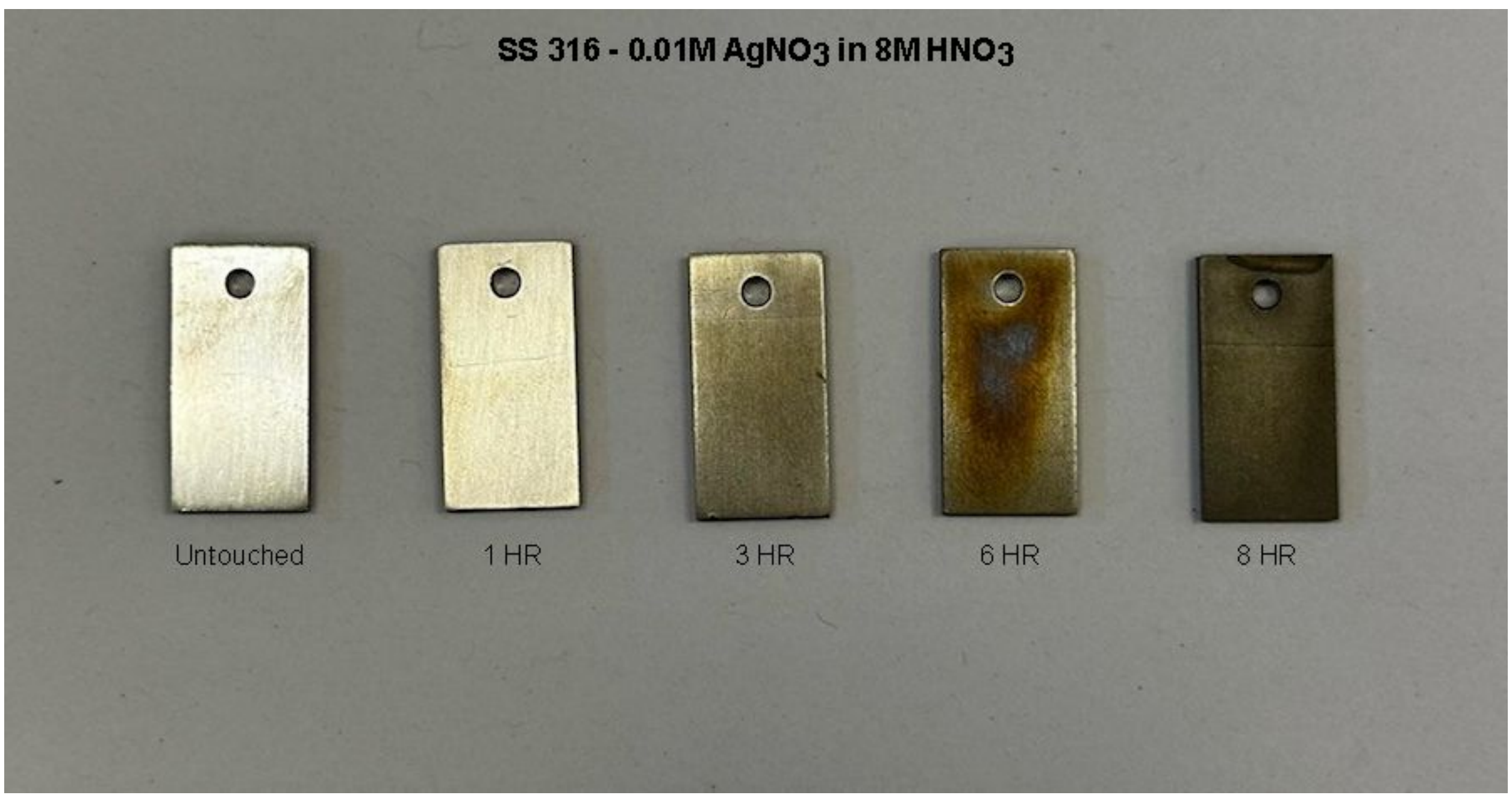

Figure S3. Picture of the pristine 316 stainless steel and coupons reacted with Ag(**II**). Samples were pulled at 1, 3, 6, and 8 hours. Conditions: initial $AgNO_3$ stock = 0.01M $AgNO_3$ concentration in 8M HNO_3, catholyte solution = 8M HNO_3.

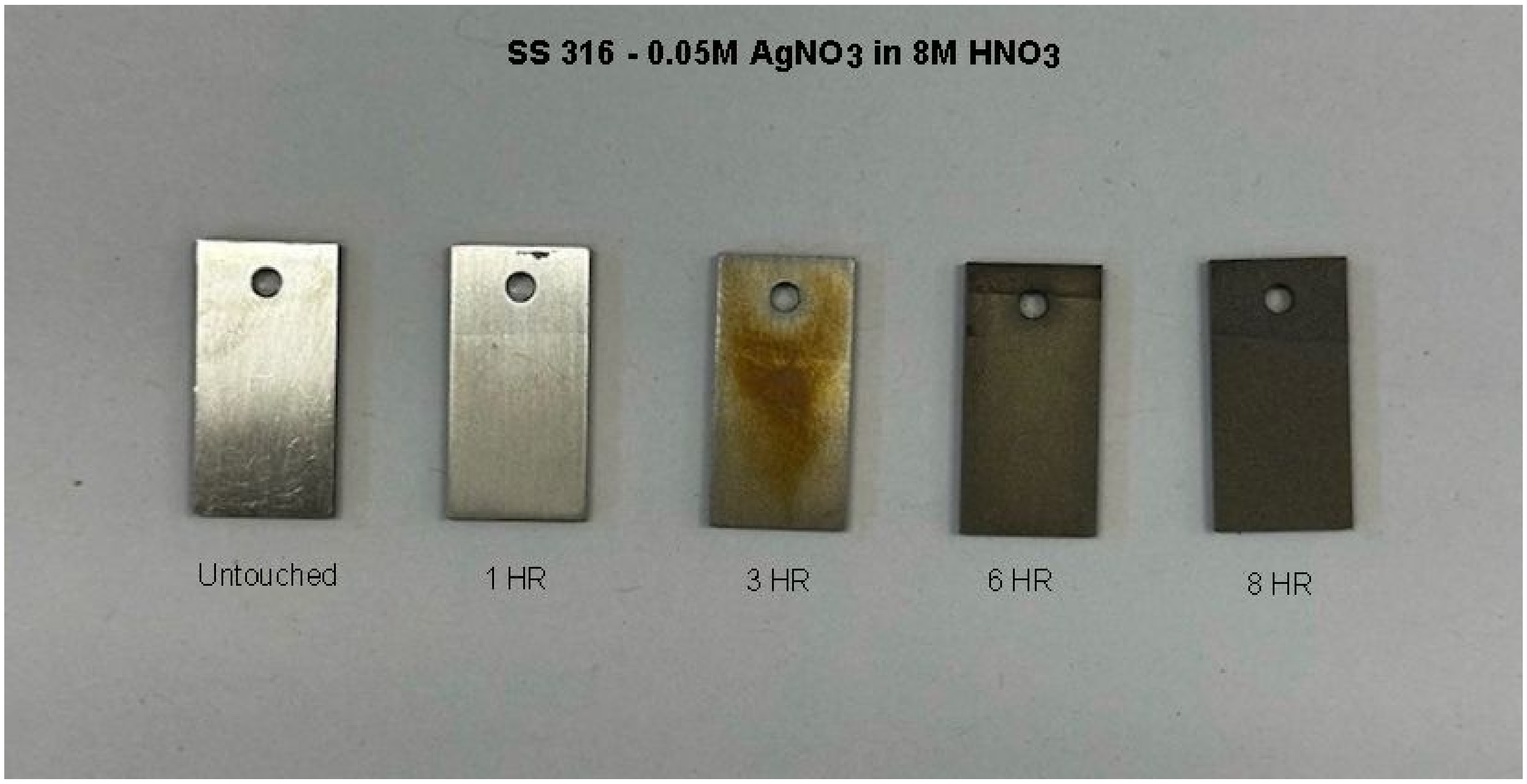

Figure S4. Picture of the pristine 316 stainless steel and coupons reacted with Ag(**II**). Samples were pulled at 1, 3, 6, and 8 hours. Conditions: initial $AgNO_3$ stock = 0.05M $AgNO_3$ concentration in 8M HNO_3, catholyte solution = 8M HNO_3.

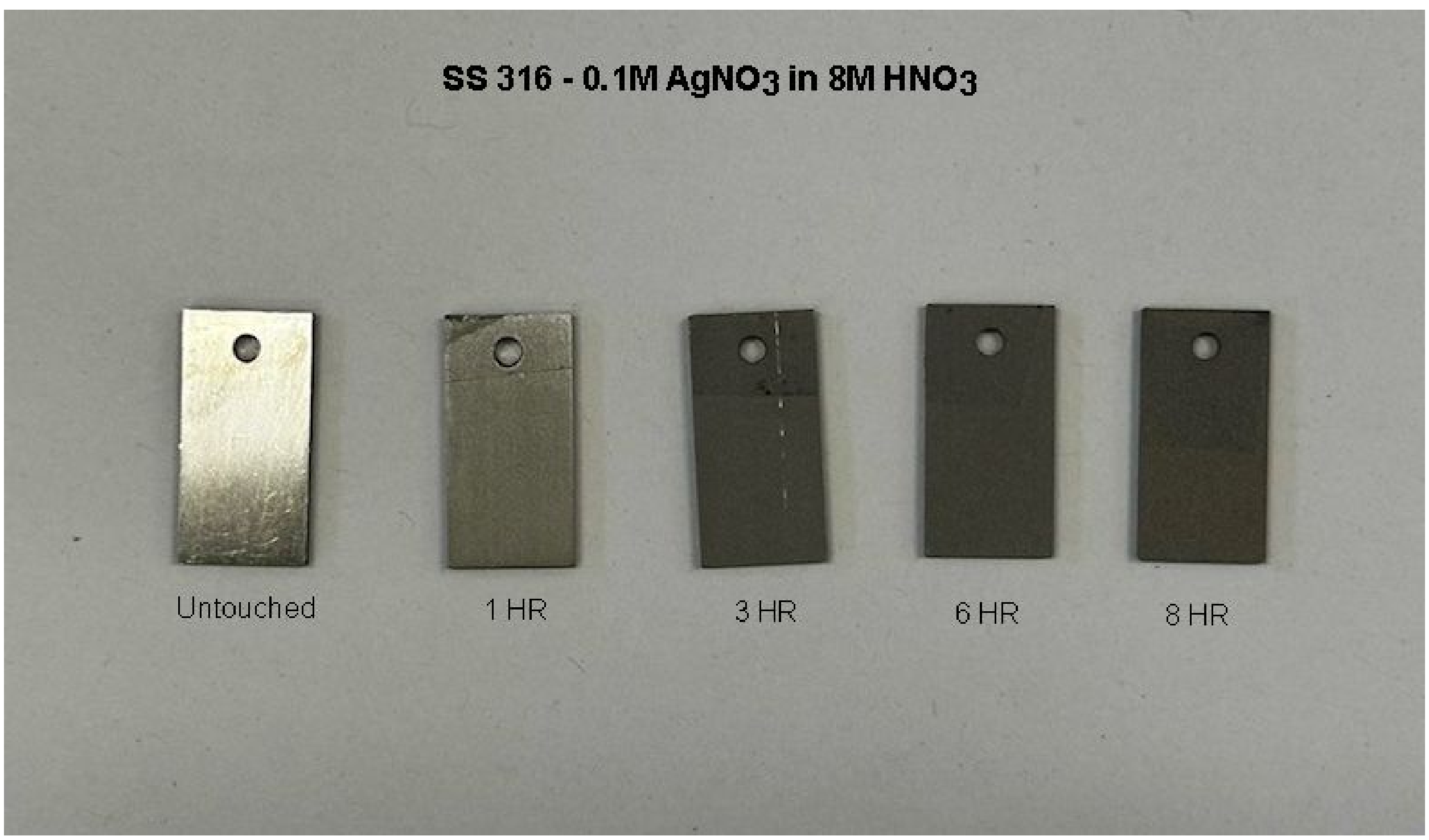

Figure S5. Picture of the pristine 316 stainless steel and coupons reacted with Ag(**II**). Samples were pulled at 1, 3, 6, and 8 hours. Conditions: initial $AgNO_3$ stock = 0.1M $AgNO_3$ concentration in 8M HNO_3, catholyte solution = 8M HNO_3.

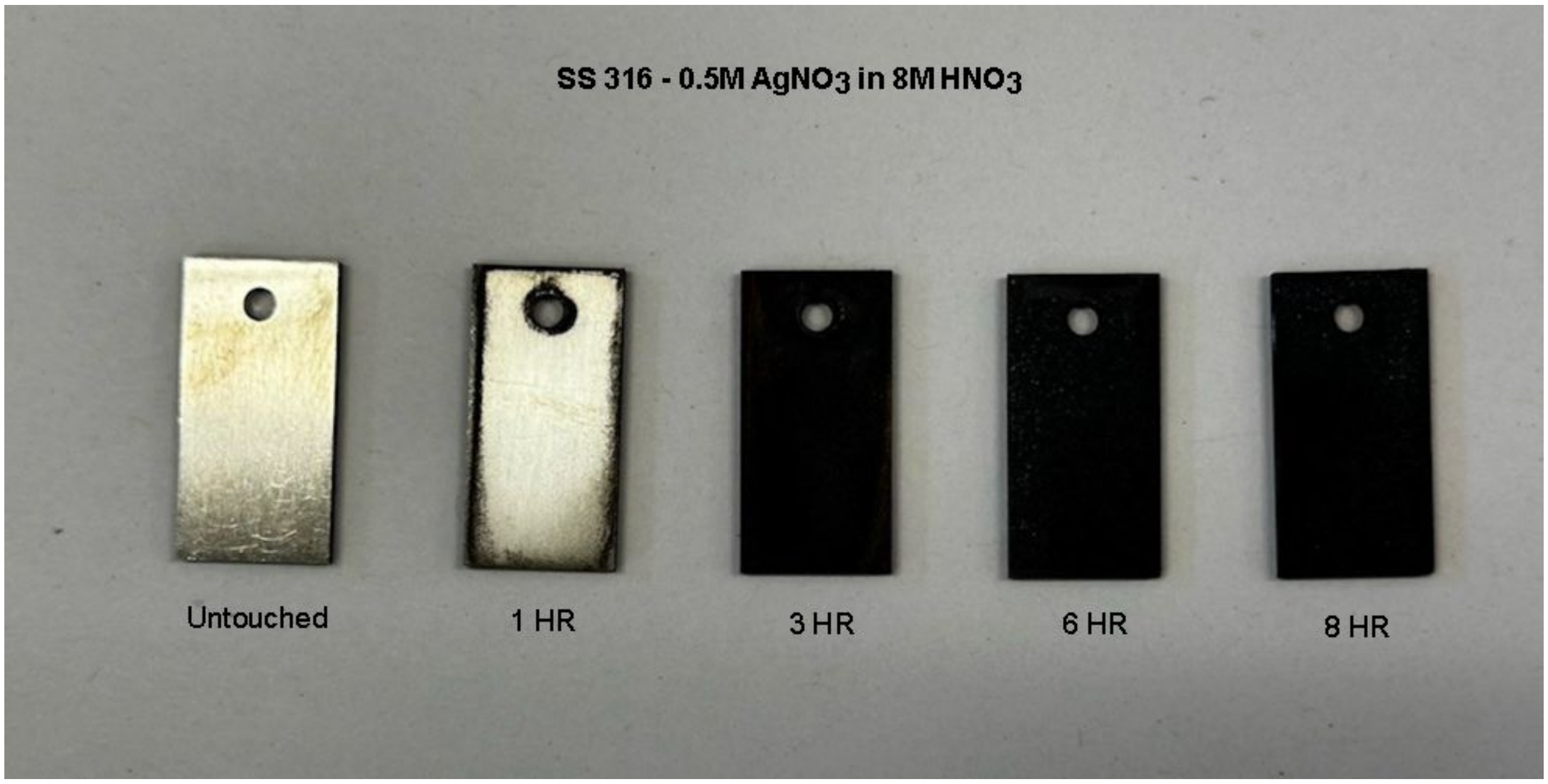

Figure S6. Picture of the pristine 316 stainless steel and coupons reacted with Ag(II). Samples were pulled at 1, 3, 6, and 8 hours. Conditions: initial $AgNO_3$ stock = 0.5M $AgNO_3$ concentration in 8M HNO_3, catholyte solution = 8M HNO_3.

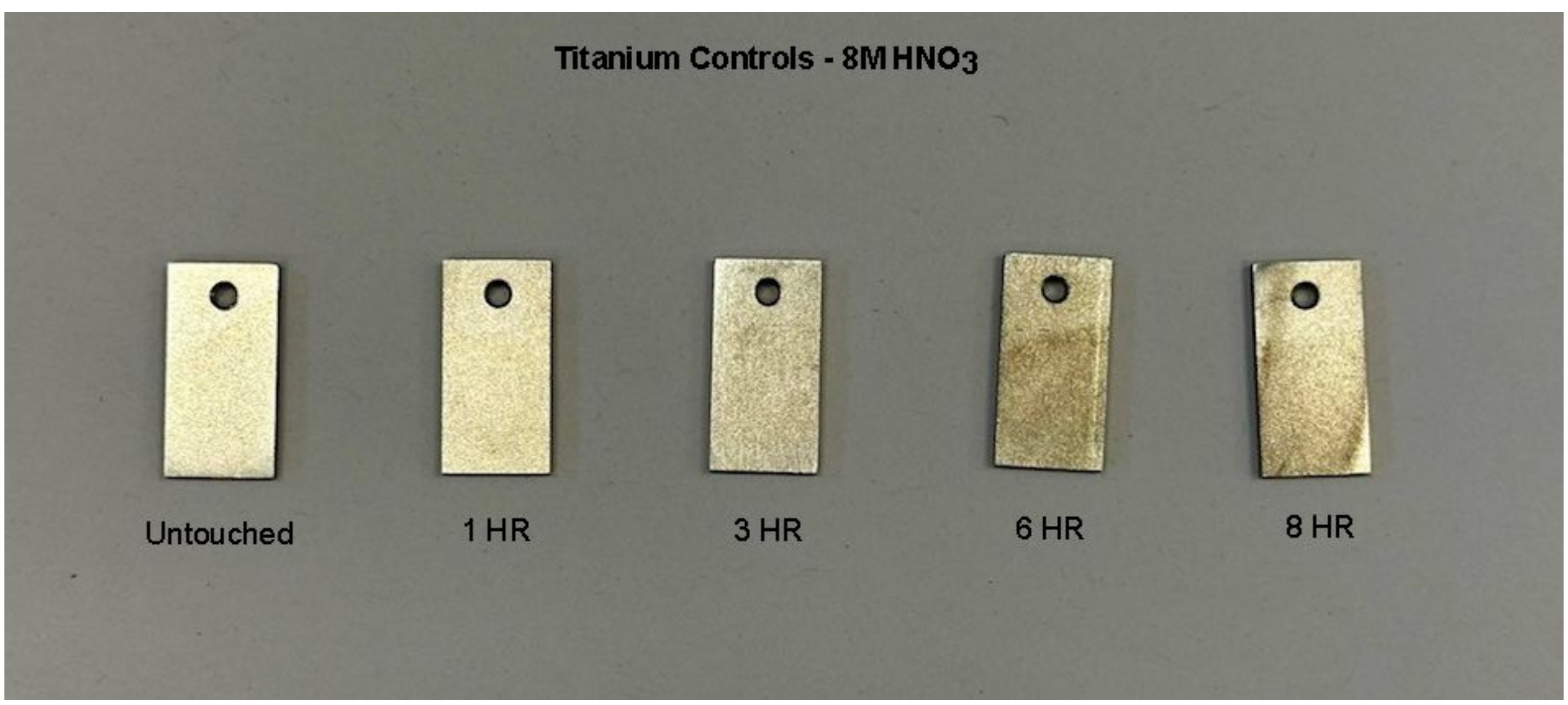

Figure S7. Picture of the pristine titanium and control coupons in nitric acid. Control samples were pulled at 1, 3, 6, and 8 hours. Conditions: 8M HNO_3

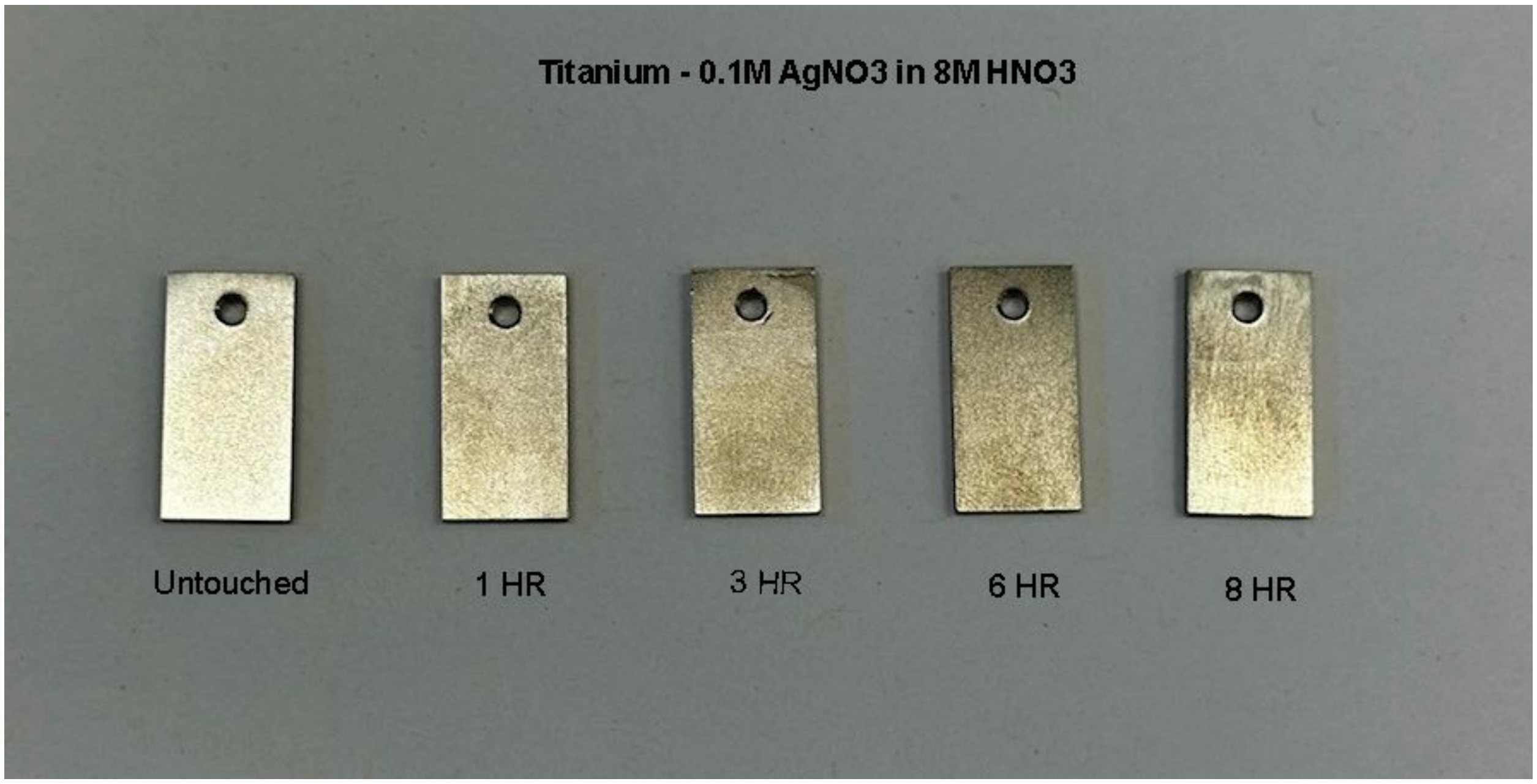

Figure S8. Picture of the pristine titanium and coupons reacted with Ag(II). Samples were pulled at 1, 3, 6, and 8 hours. Conditions: initial $AgNO_3$ stock = 0.1M $AgNO_3$ concentration in 8M HNO_3, catholyte solution = 8M HNO_3.

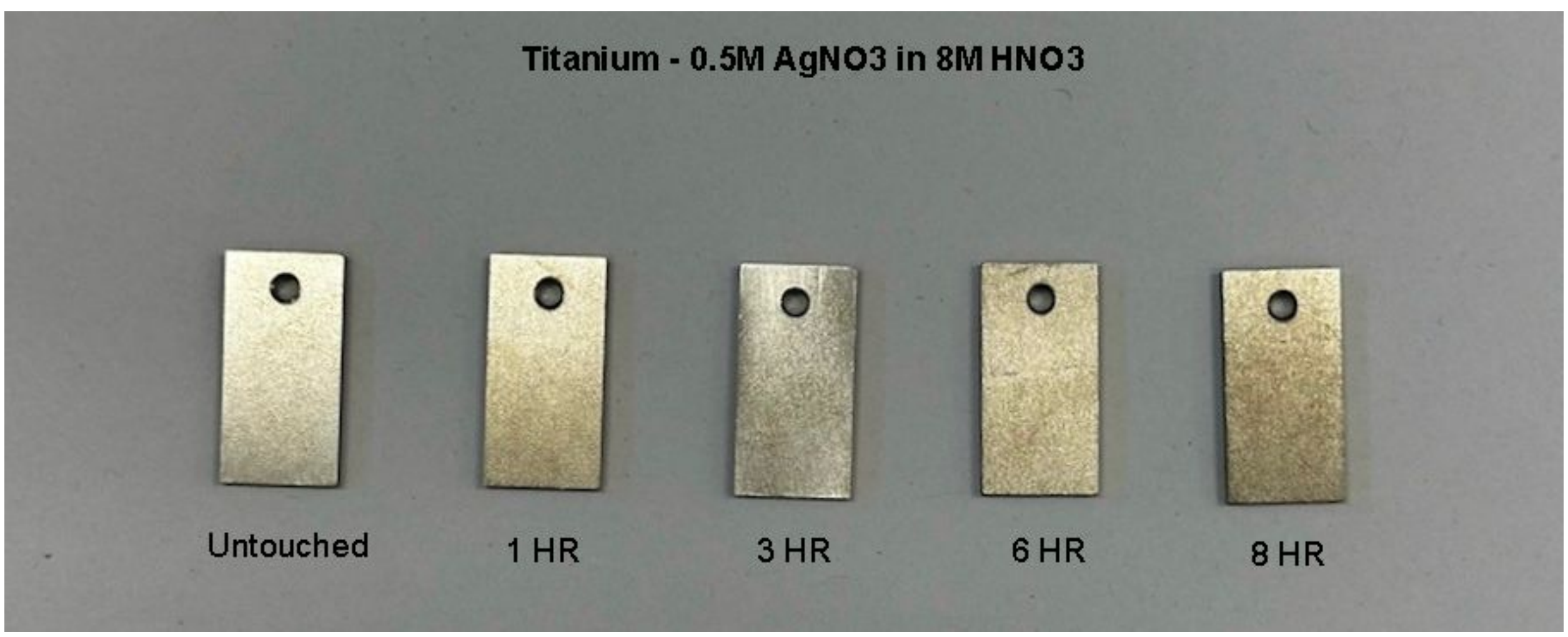

Figure S9. Picture of the pristine titanium and coupons reacted with Ag(II). Samples were pulled at 1, 3, 6, and 8 hours. Conditions: initial $AgNO_3$ stock = 0.5M $AgNO_3$ concentration in 8M HNO_3, catholyte solution = 8M HNO_3.

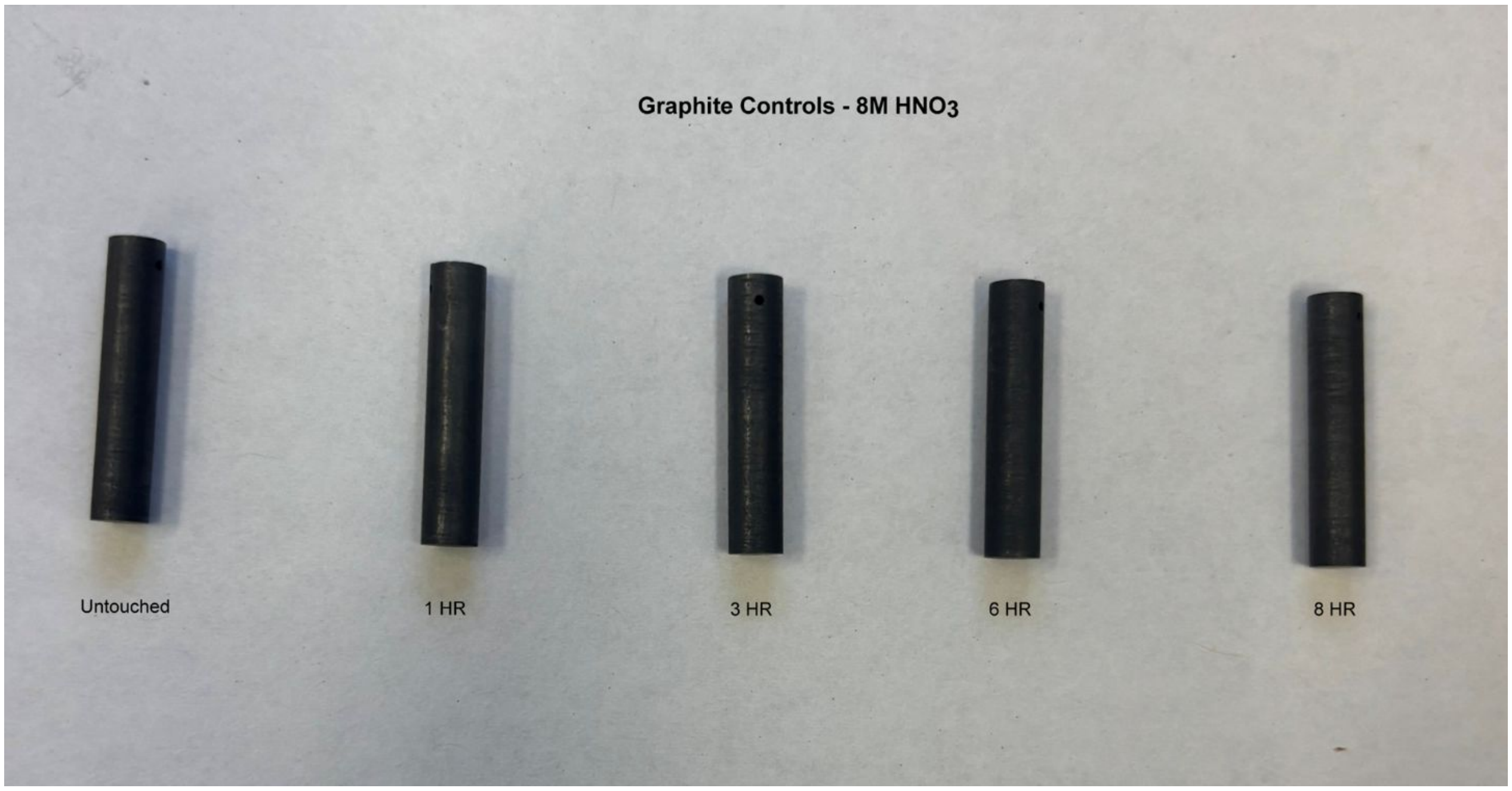

Figure S10. Picture of the pristine graphite and control coupons in nitric acid. Control samples were pulled at 1, 3, 6, and 8 hours. Conditions: 8M HNO_3

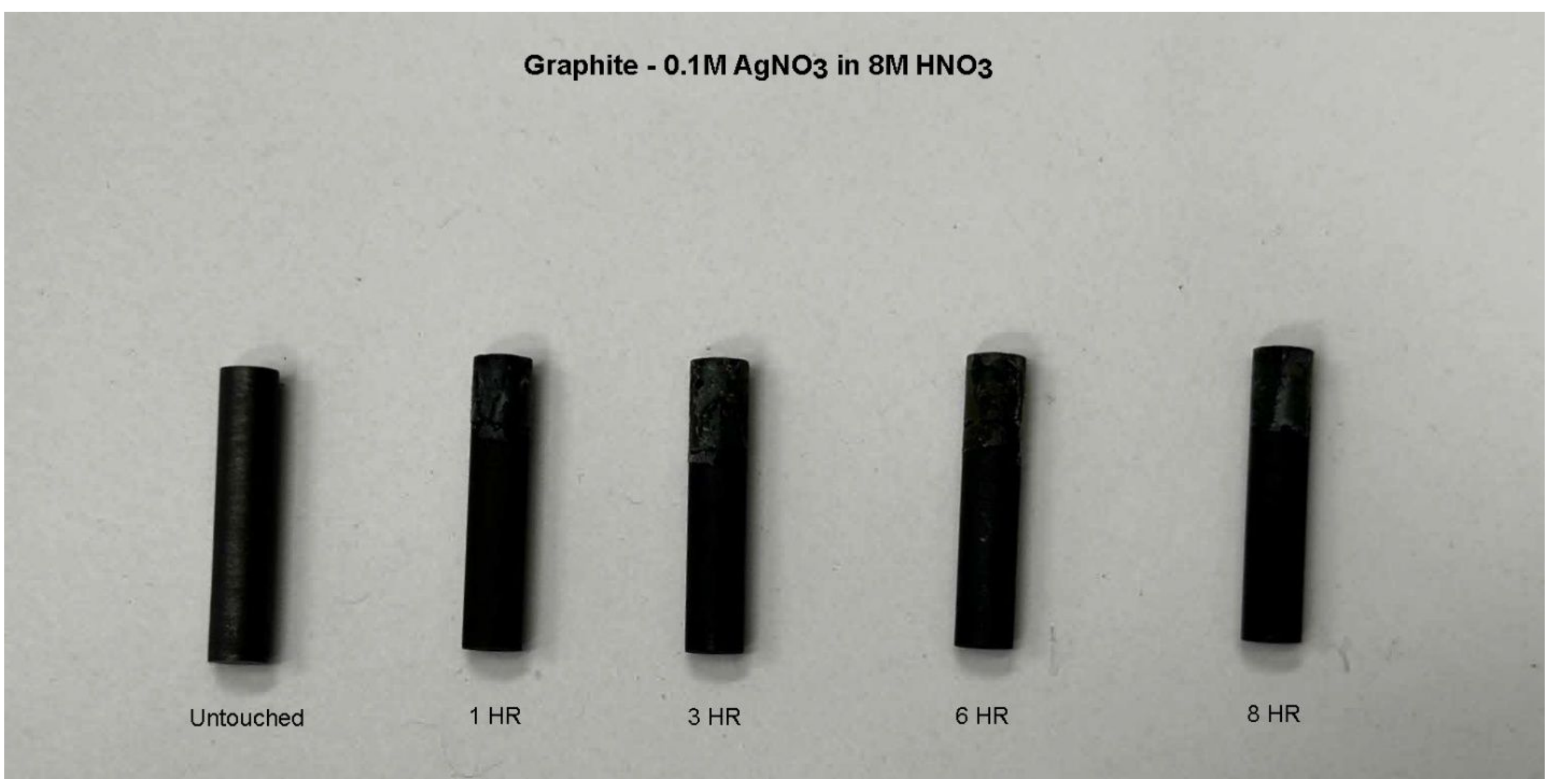

Figure S11. Picture of the pristine graphite and coupons reacted with Ag(II). Samples were pulled at 1, 3, 6, and 8 hours. Conditions: initial $AgNO_3$ stock = 0.1M $AgNO_3$ concentration in 8M HNO_3, catholyte solution = 8M HNO_3.

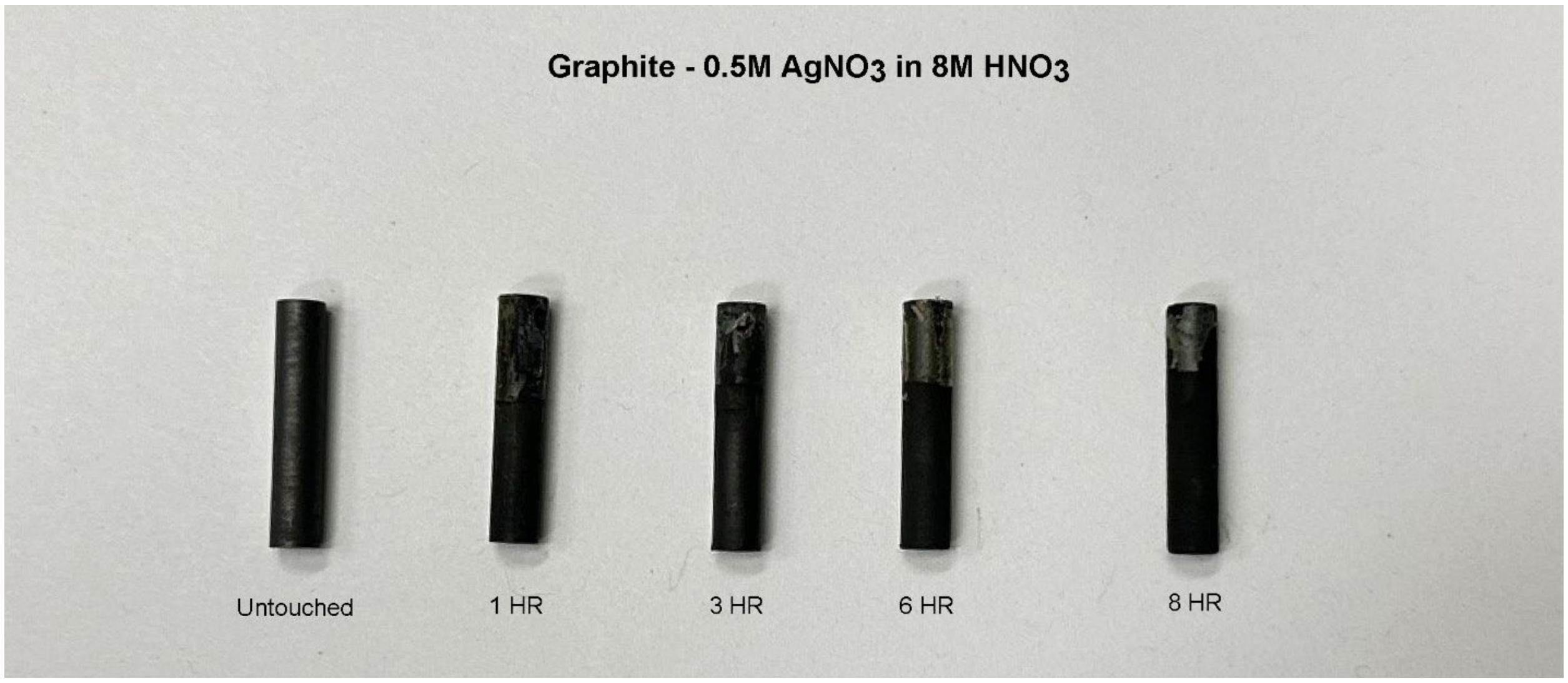

Figure S12. Picture of the pristine graphite and coupons reacted with Ag(II). Samples were pulled at 1, 3, 6, and 8 hours. Conditions: initial $AgNO_3$ stock = 0.5M $AgNO_3$ concentration in 8M HNO_3, catholyte solution = 8M HNO_3.

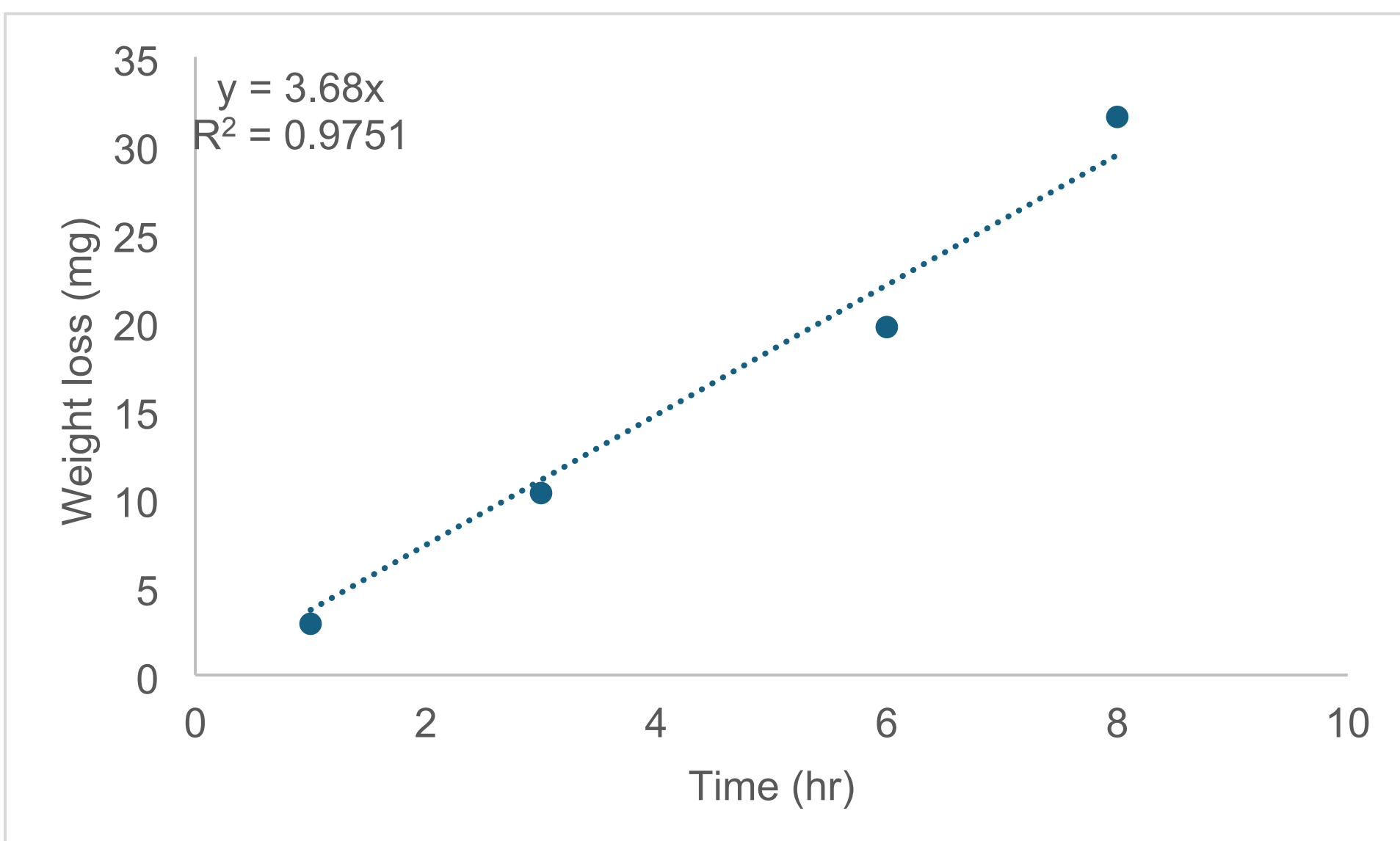

Figure S13. Dissolution rate of 316 stainless steel reacted with Ag(II). Samples were pulled at 1, 3, 6, and 8 hours. Conditions: initial $AgNO_3$ stock = 0.01M $AgNO_3$ concentration in 8M HNO_3, catholyte solution = 8M HNO_3.

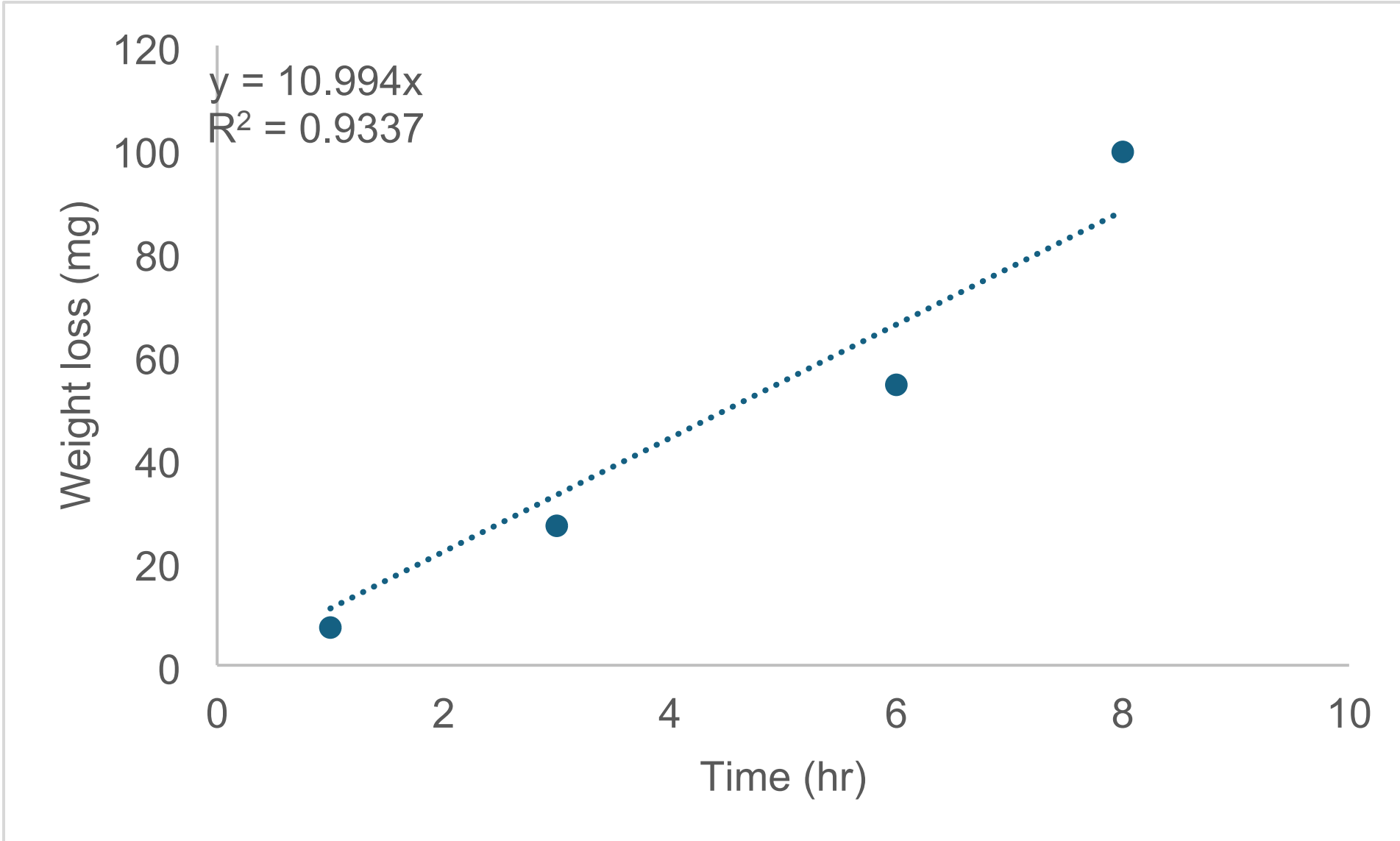

Figure S14. Dissolution rate of 316 stainless steel reacted with Ag(II). Samples were pulled at 1, 3, 6, and 8 hours. Conditions: initial $AgNO_3$ stock = 0.05M $AgNO_3$ concentration in 8M HNO_3, catholyte solution = 8M HNO_3.

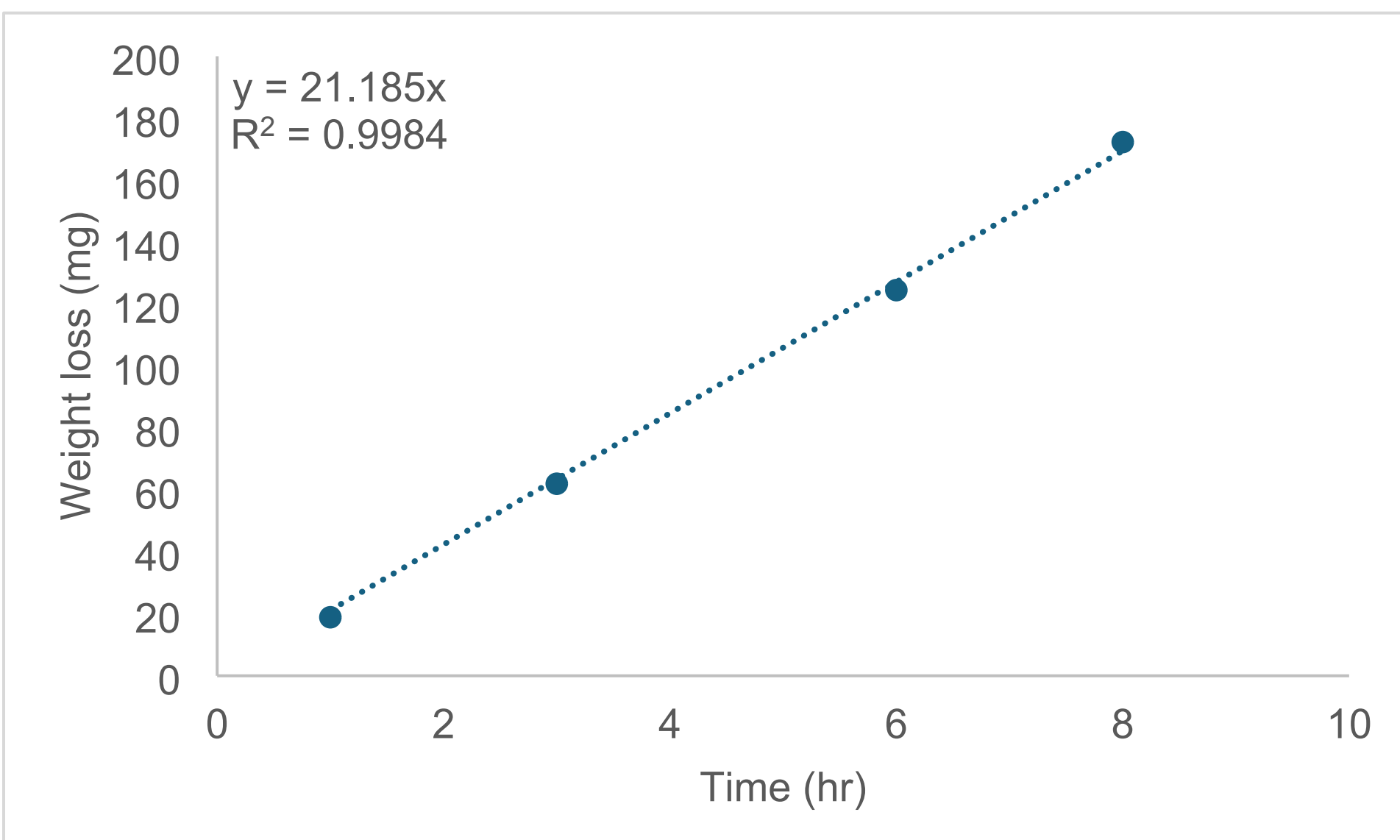

Figure S15. Dissolution rate of 316 stainless steel reacted with Ag(II). Samples were pulled at 1, 3, 6, and 8 hours. Conditions: initial $AgNO_3$ stock = 0.1M $AgNO_3$ concentration in 8M HNO_3, catholyte solution = 8M HNO_3.

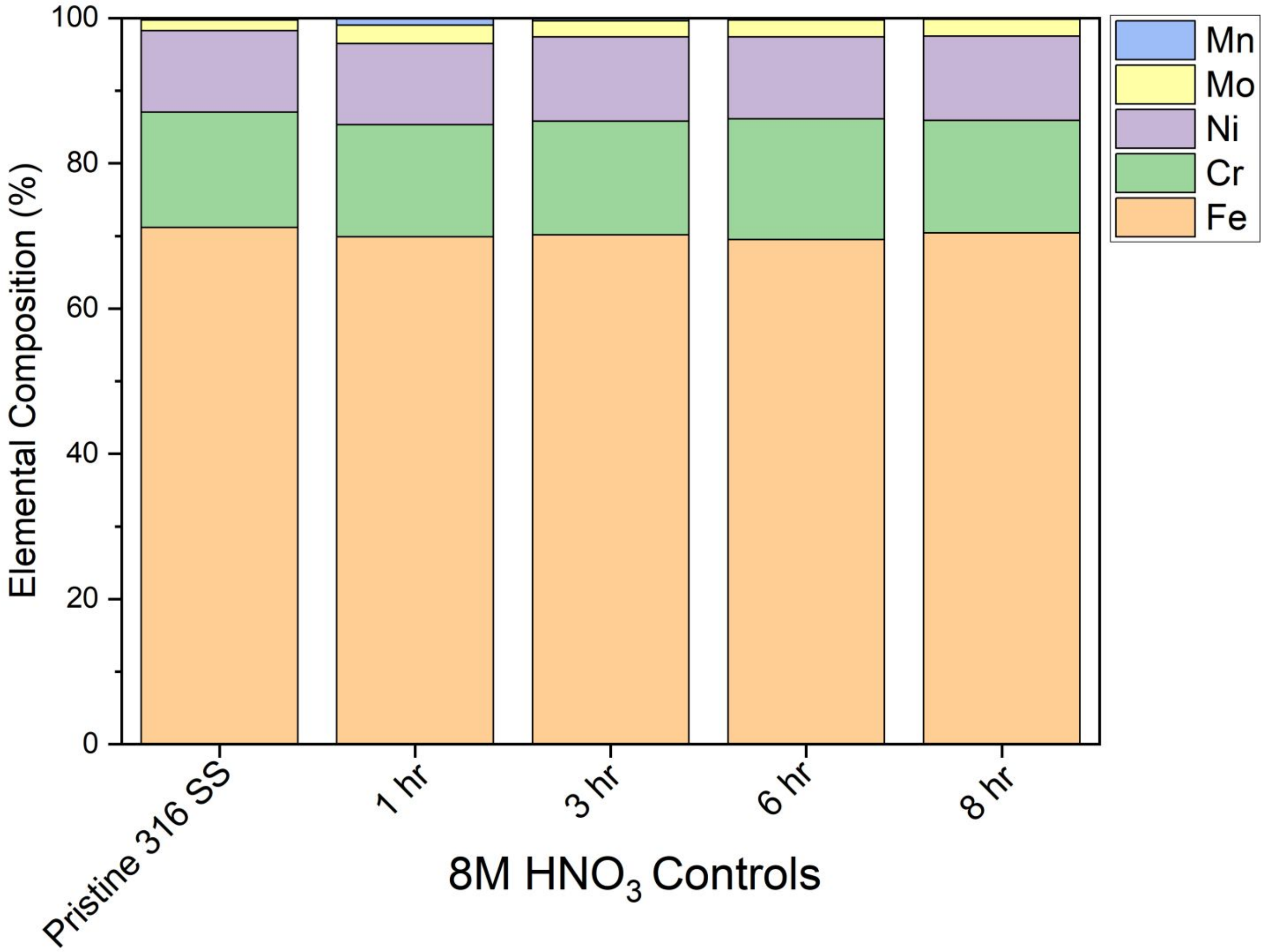

Figure S16. Surface elemental composition of pristine 316 stainless steel and coupons in nitric acid. Control samples were pulled at 1, 3, 6, and 8 hours and determined by SEM/EDX analysis. Conditions: 8M HNO_3.

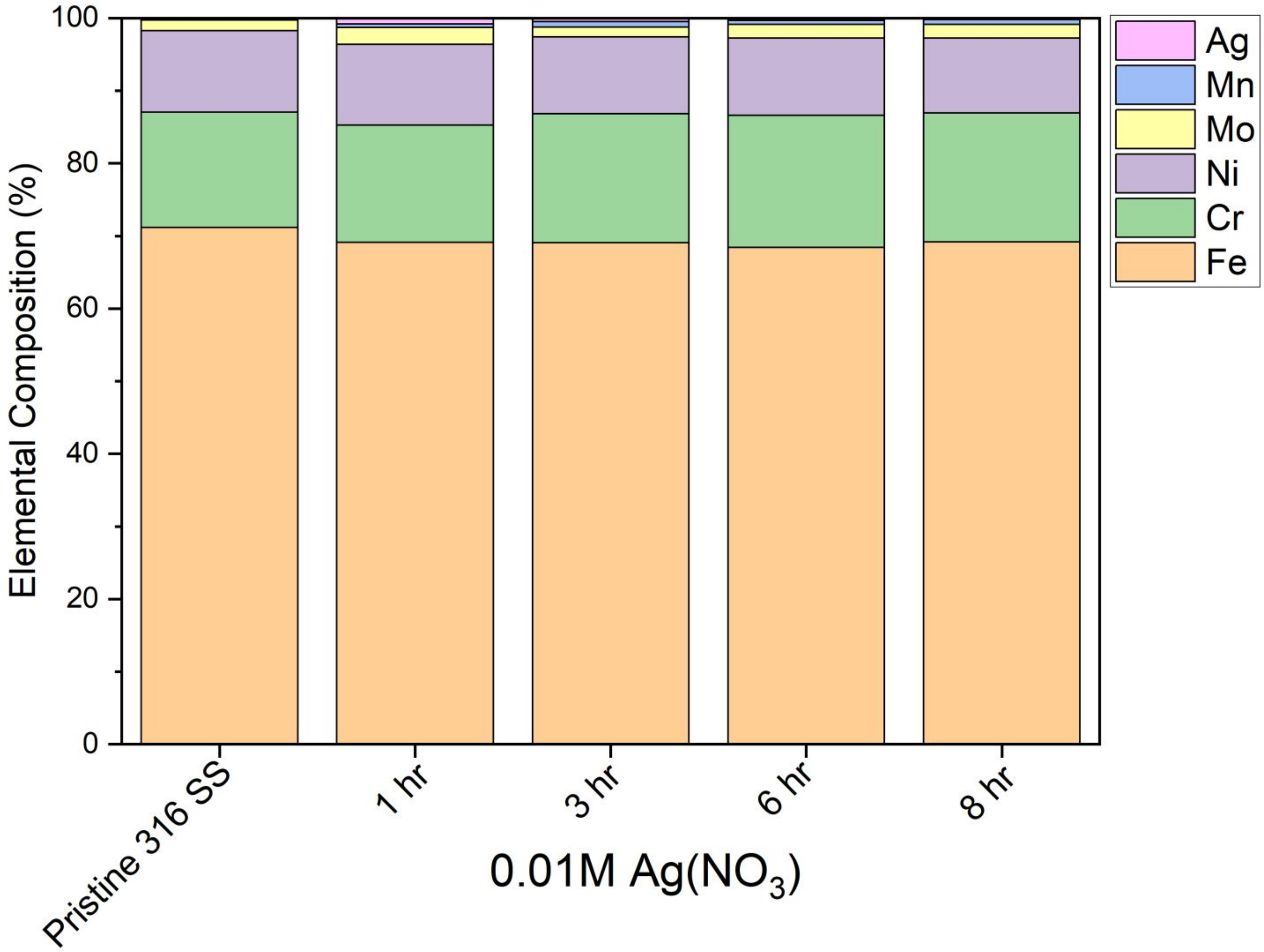

Figure S17. Surface elemental composition of pristine 316 stainless steel and coupons reacted with Ag(II). Samples were pulled at 1, 3, 6, and 8 hours and determined by SEM/EDX analysis. Conditions: initial $AgNO_3$ stock = 0.01M $AgNO_3$ concentration in 8M HNO_3, catholyte solution = 8M HNO_3.

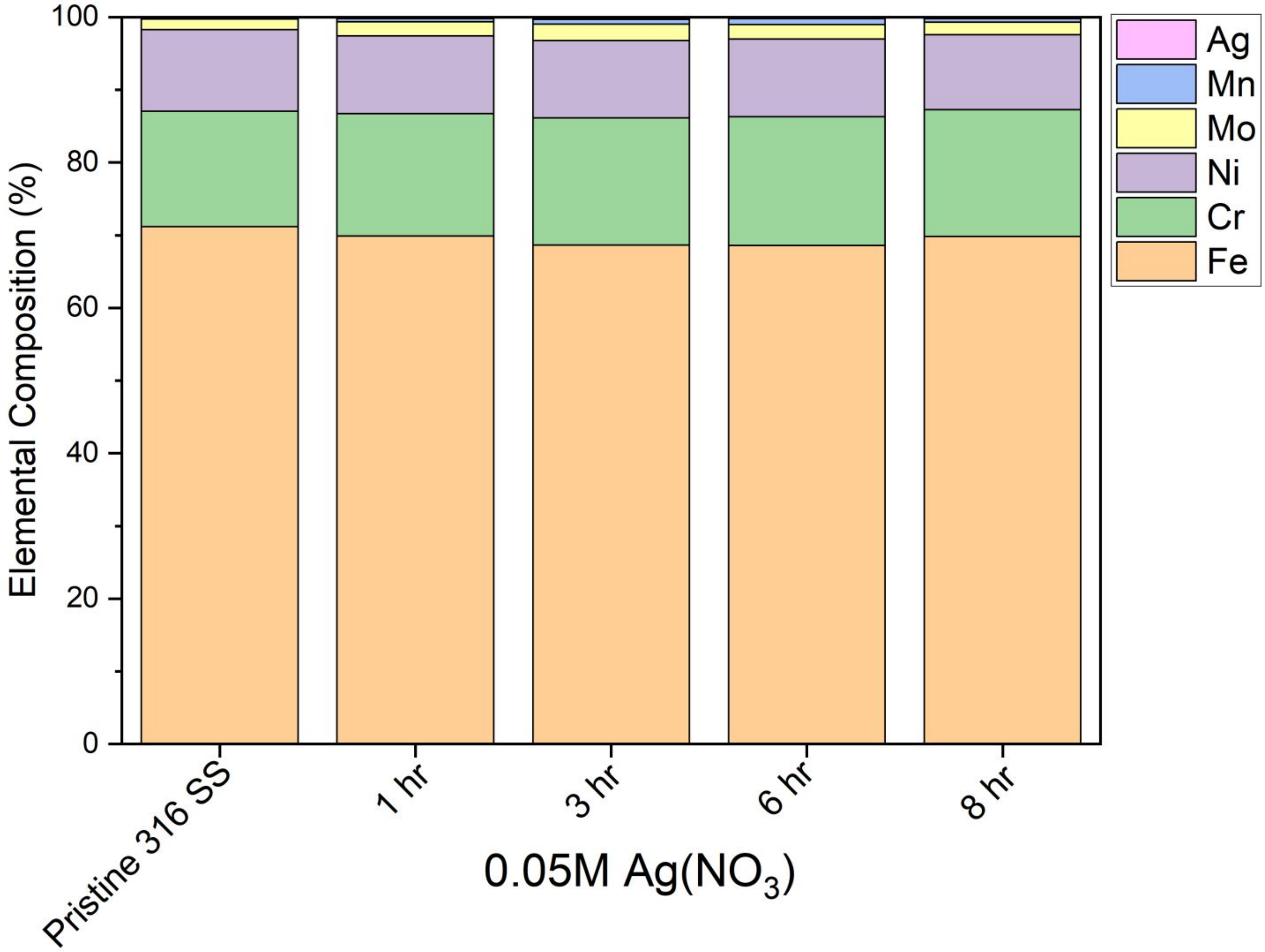

Figure S18. Surface elemental composition of pristine 316 stainless steel and coupons reacted with Ag(II). Samples were pulled at 1, 3, 6, and 8 hours and determined by SEM/EDX analysis. Conditions: initial $AgNO_3$ stock = 0.05M $AgNO_3$ concentration in 8M HNO_3, catholyte solution = 8M HNO_3.

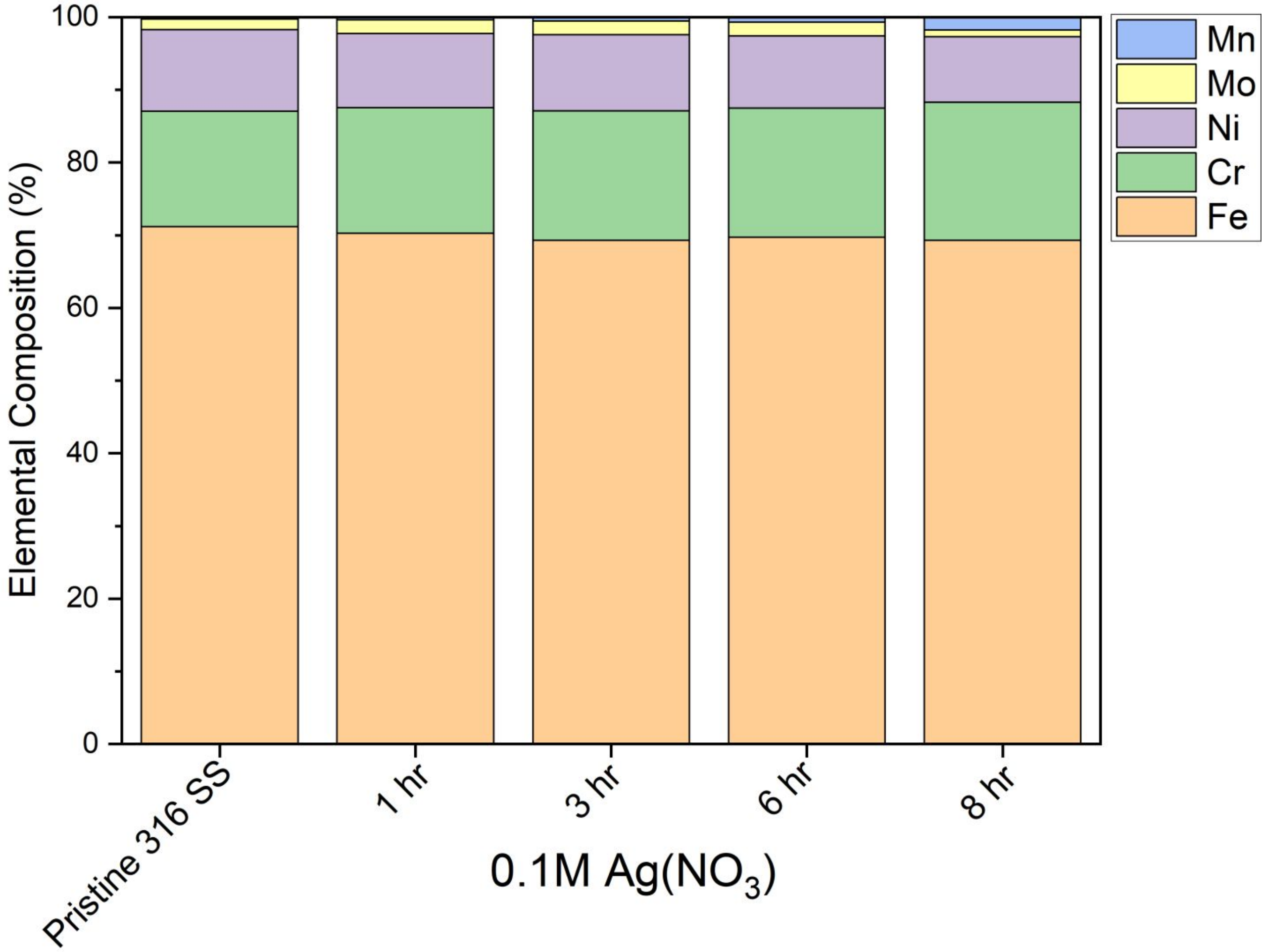

Figure S19. Surface elemental composition of pristine 316 stainless steel and coupons reacted with Ag(II). Samples were pulled at 1, 3, 6, and 8 hours and determined by SEM/EDX analysis. Conditions: initial $AgNO_3$ stock = 0.1M $AgNO_3$ concentration in 8M HNO_3, catholyte solution = 8M HNO_3.

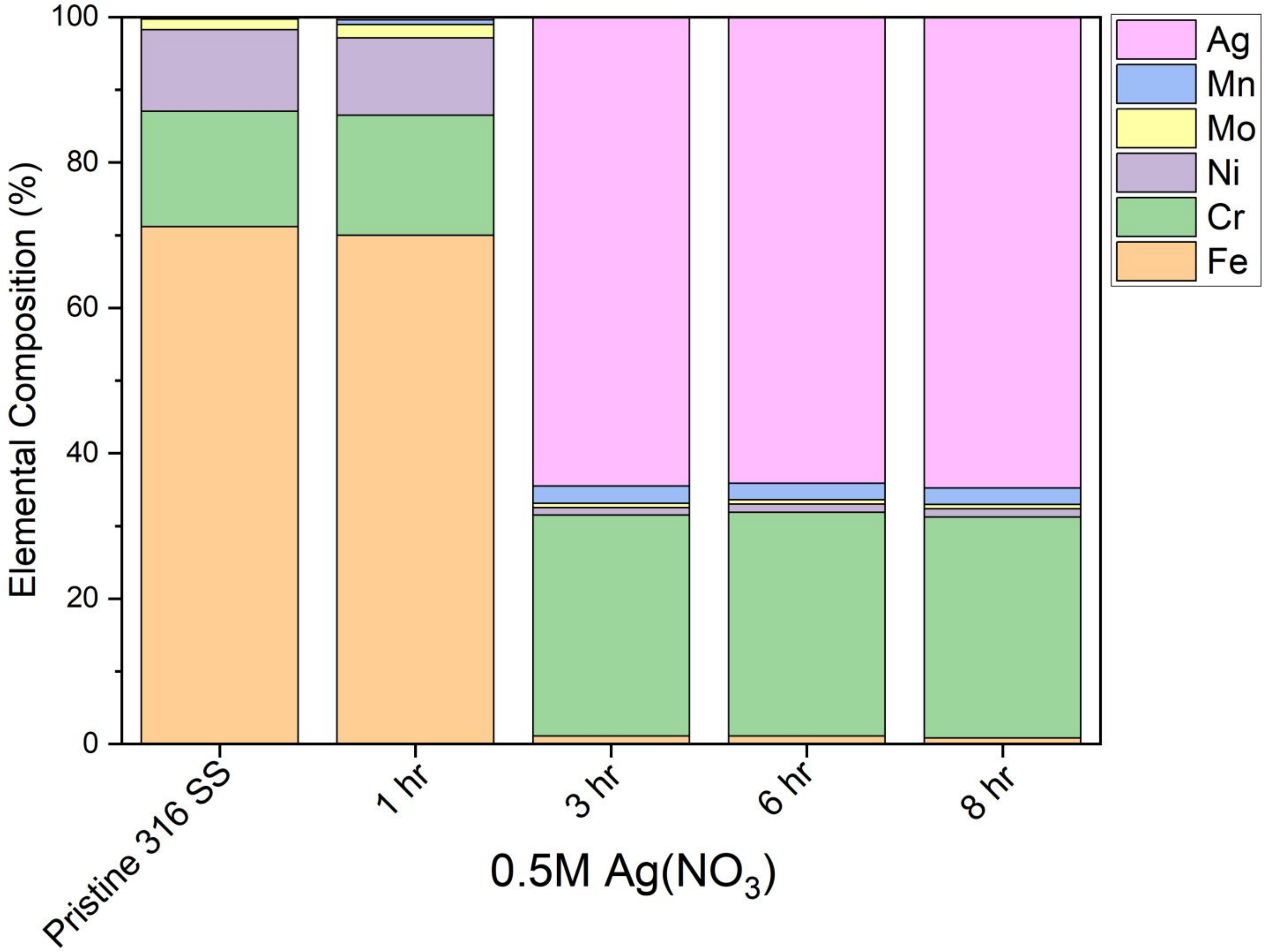

Figure S20. Surface elemental composition of pristine 316 stainless steel and coupons reacted with Ag(II). Samples were pulled at 1, 3, 6, and 8 hours and determined by SEM/EDX analysis. Conditions: initial $AgNO_3$ stock = 0.5M $AgNO_3$ concentration in 8M HNO_3, catholyte solution = 8M HNO_3.

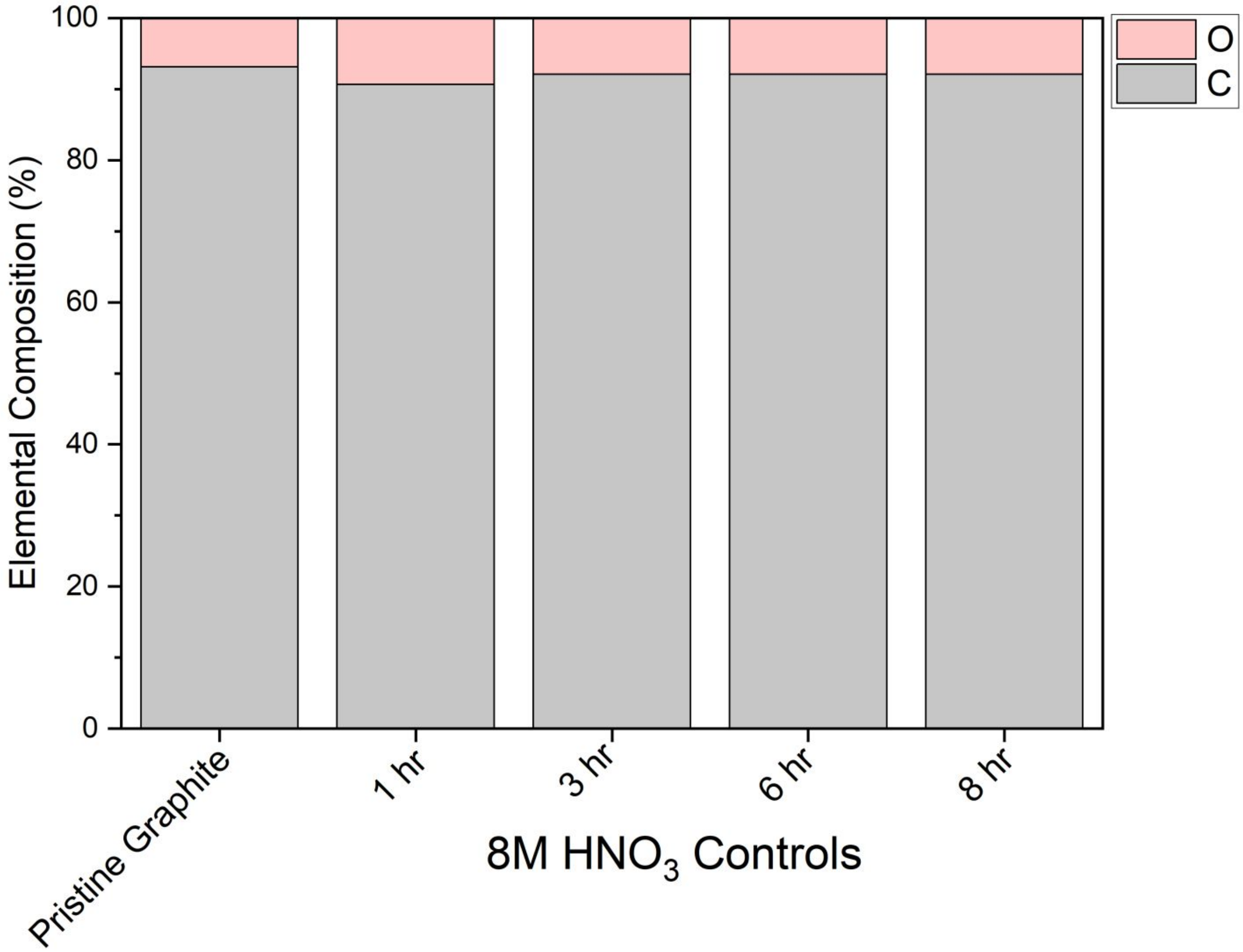

Figure S21. Surface elemental composition of pristine graphite and graphite coupons in nitric acid. Control samples were pulled at 1, 3, 6, and 8 hours and determined by SEM/EDX analysis. Conditions: 8M HNO_3.

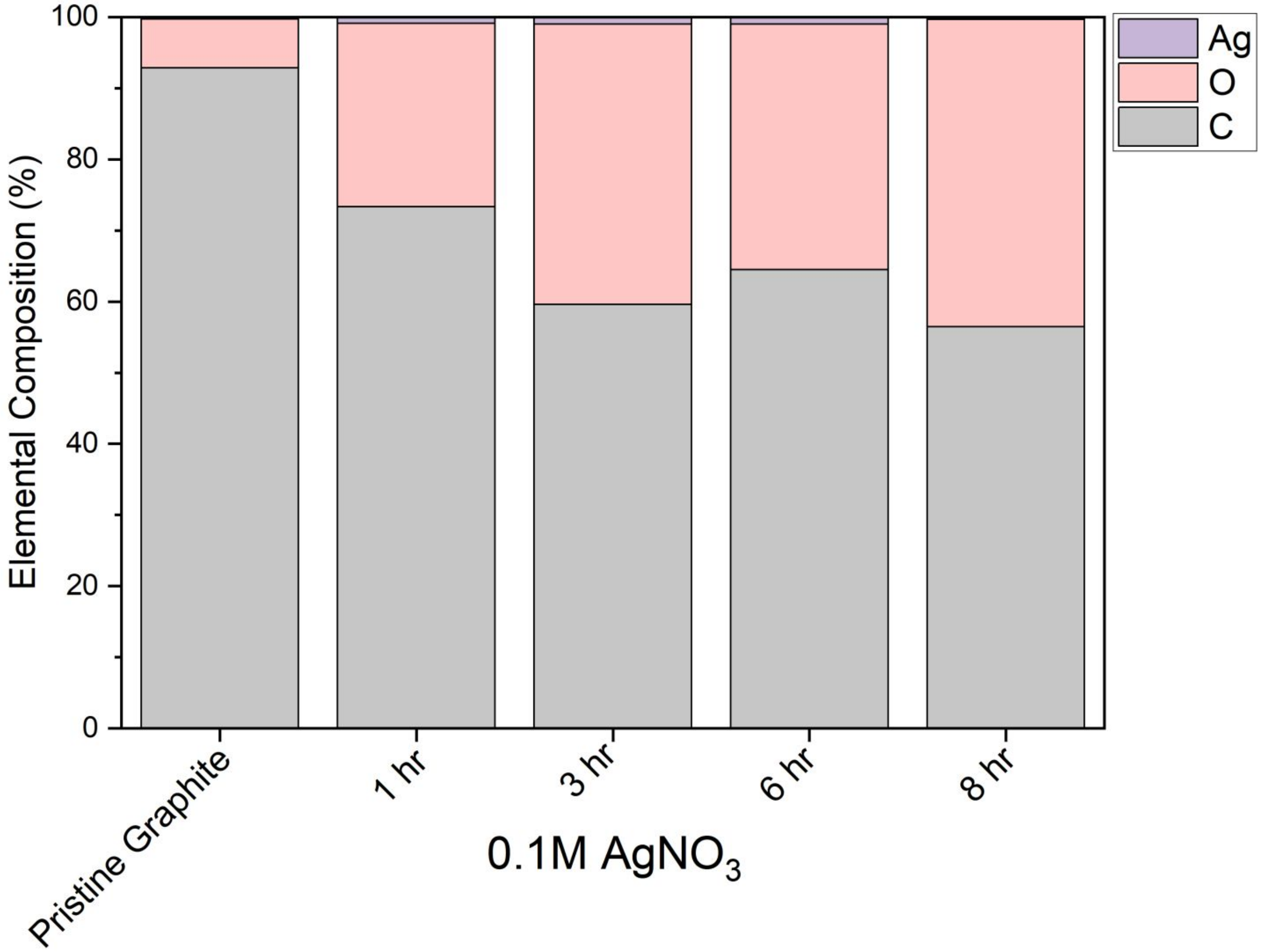

Figure S22. Surface elemental composition of pristine graphite and graphite coupons reacted with Ag(II). Samples were pulled at 1, 3, 6, and 8 hours and determined by SEM/EDX analysis. Conditions: initial $AgNO_3$ stock = 0.1M $AgNO_3$ concentration in 8M HNO_3, catholyte solution = 8M HNO_3.

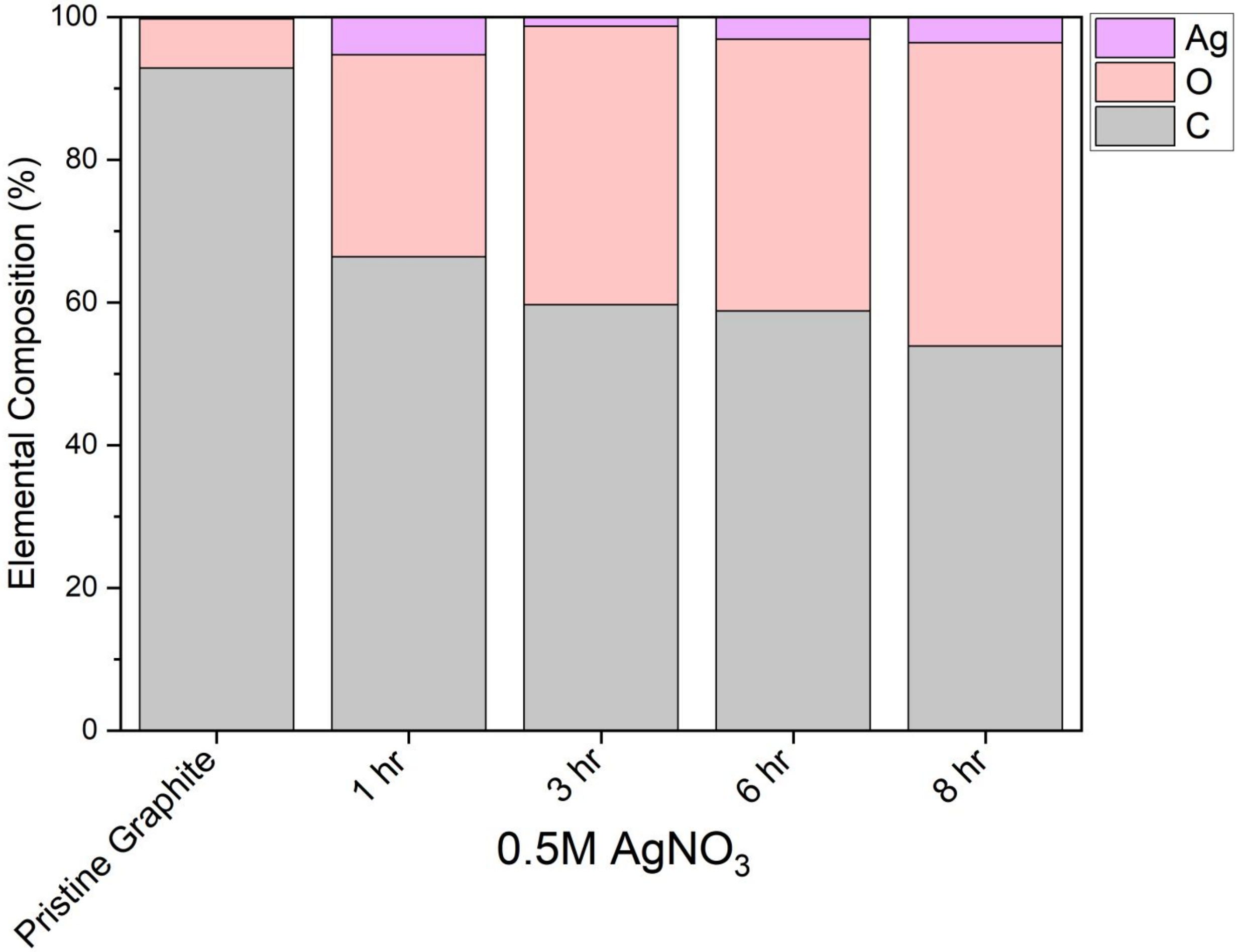

Figure S23. Surface elemental composition of pristine graphite and graphite coupons reacted with Ag(II). Samples were pulled at 1, 3, 6, and 8 hours and determined by SEM/EDX analysis. Conditions: initial $AgNO_3$ stock = 0.5M $AgNO_3$ concentration in 8M HNO_3, catholyte solution = 8M HNO_3.

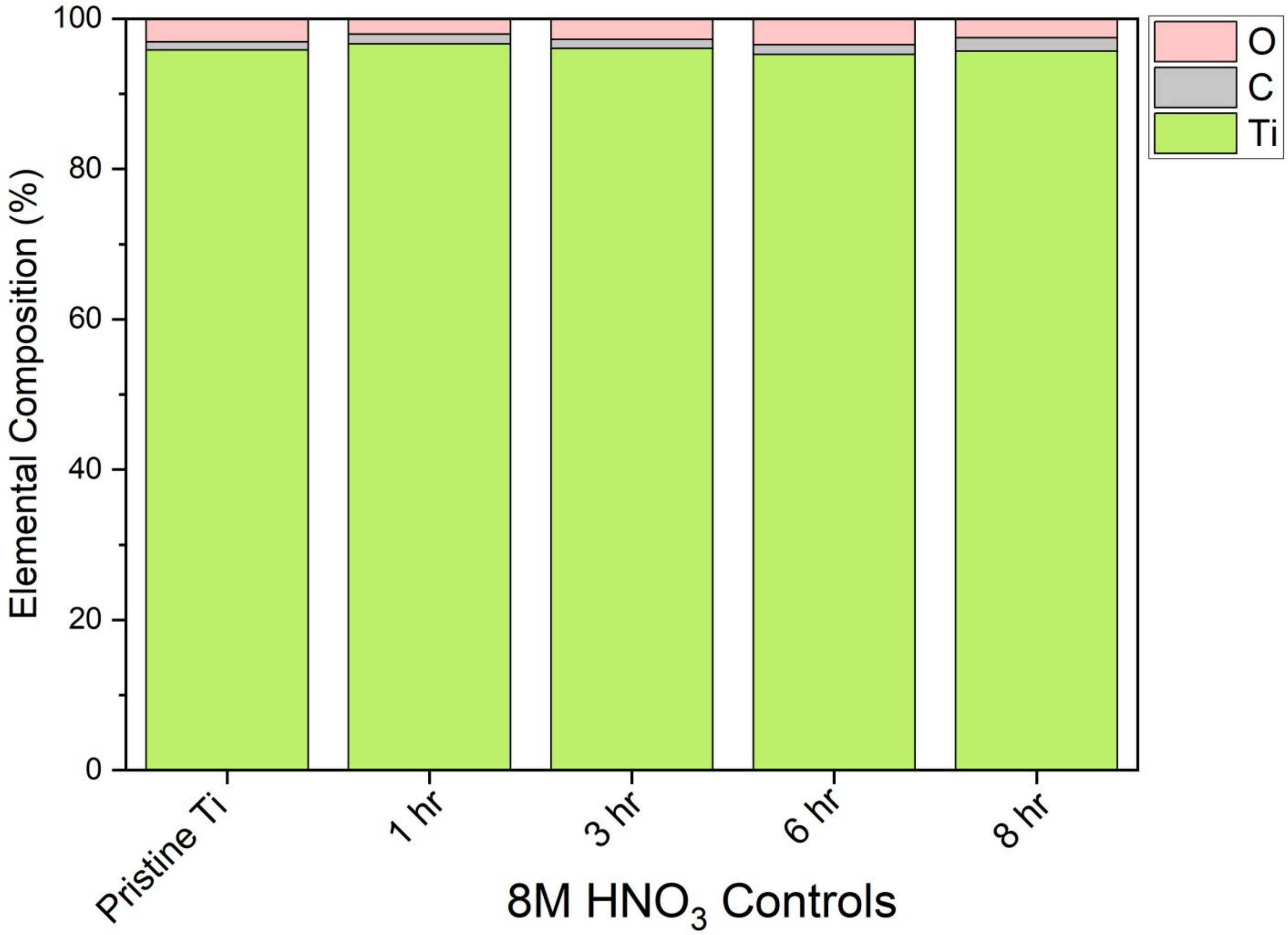

Figure S24. Surface elemental composition of pristine titanium and titanium coupons in nitric acid. Control samples were pulled at 1, 3, 6, and 8 hours and determined by SEM/EDX analysis. Conditions: 8M HNO_3.

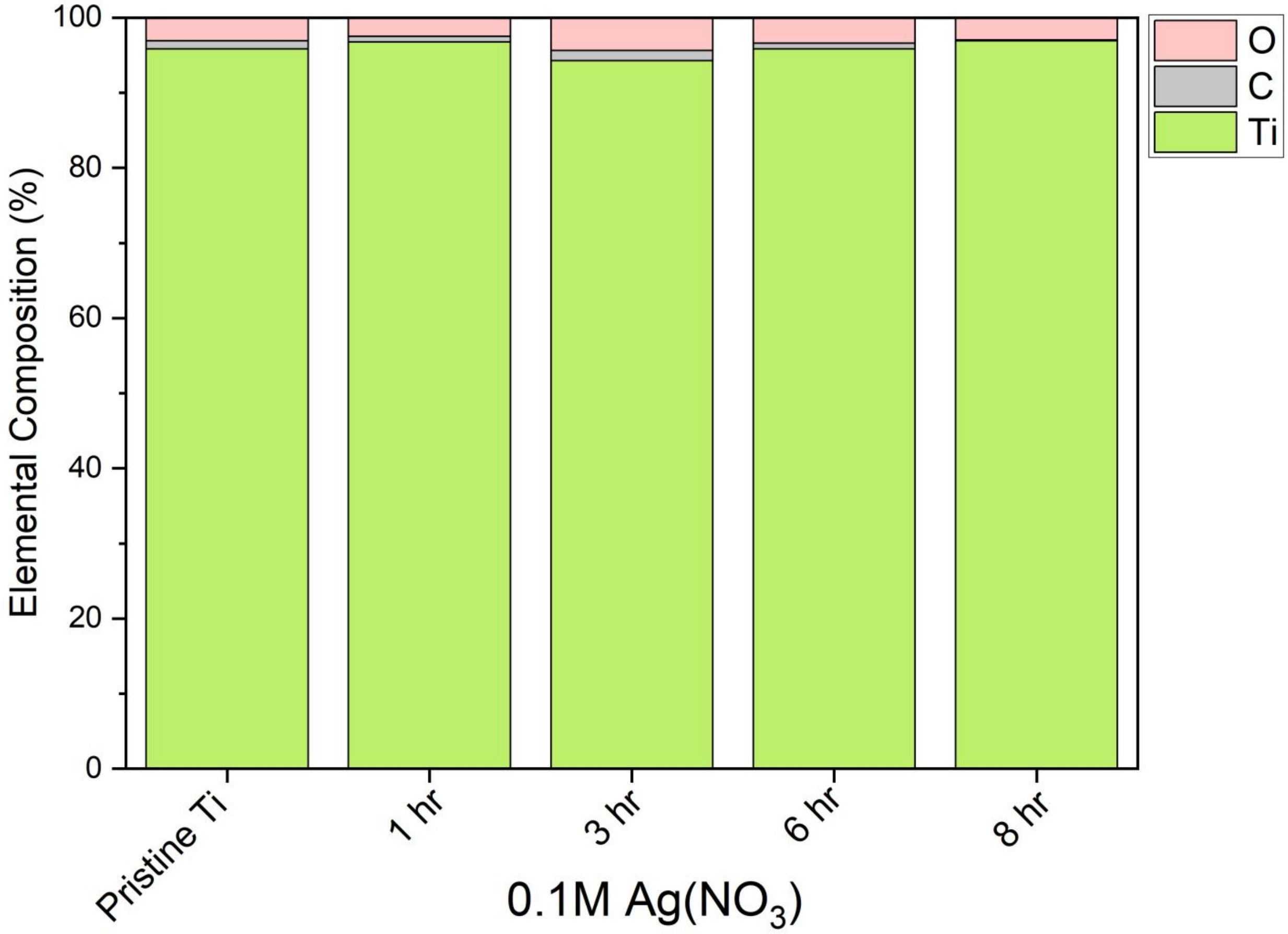

Figure S25. Surface elemental composition of pristine titanium and titanium coupons reacted with Ag(II). Samples were pulled at 1, 3, 6, and 8 hours and determined by SEM/EDX analysis. Conditions: initial $AgNO_3$ stock = 0.1M $AgNO_3$ concentration in 8M HNO_3, catholyte solution = 8M HNO_3.

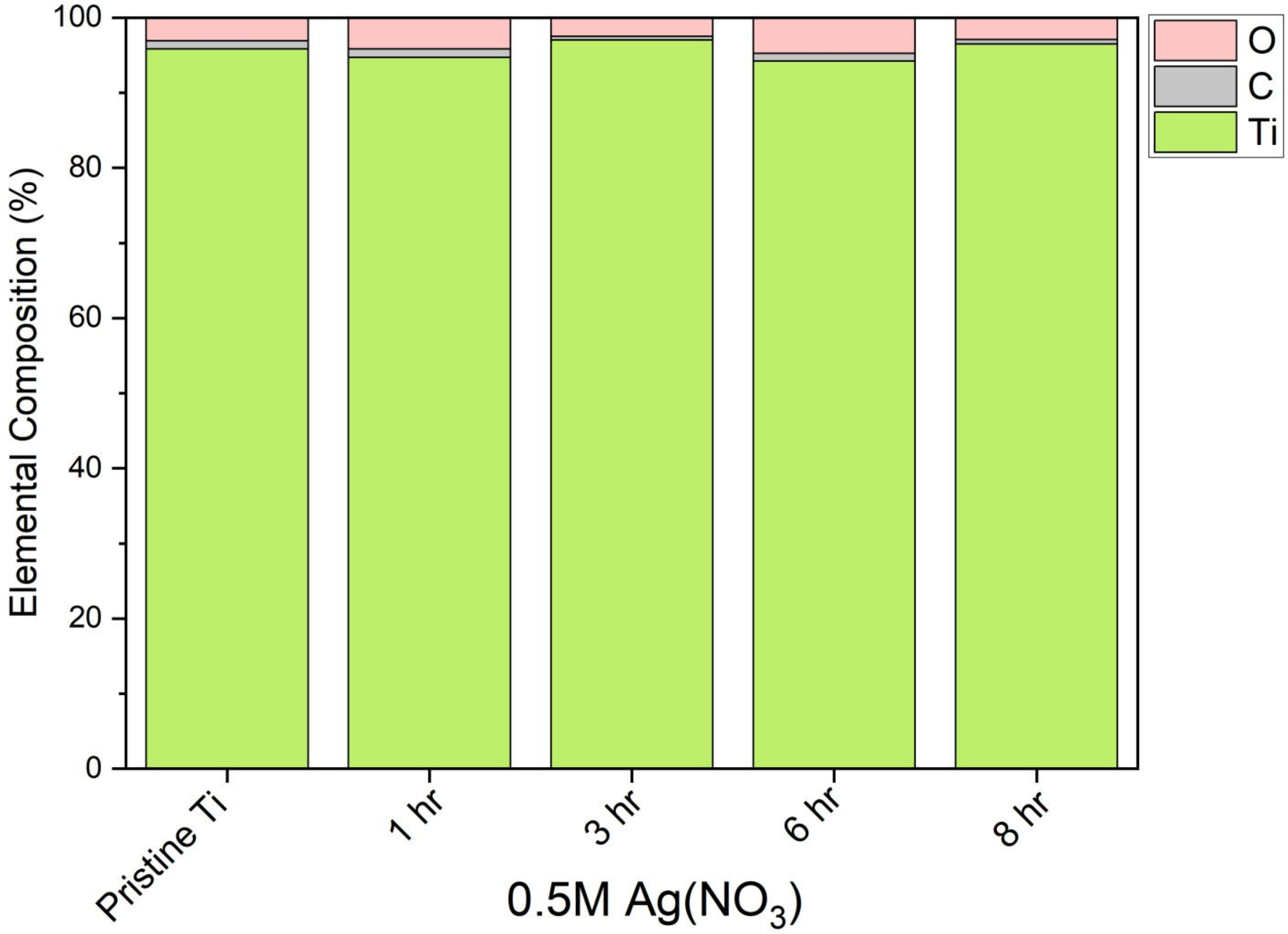

Figure S26. Surface elemental composition of pristine titanium and titanium coupons reacted with Ag(II). Samples were pulled at 1, 3, 6, and 8 hours and determined by SEM/EDX analysis. Conditions: initial $AgNO_3$ stock = 0.5M $AgNO_3$ concentration in 8M HNO_3, catholyte solution = 8M HNO_3.

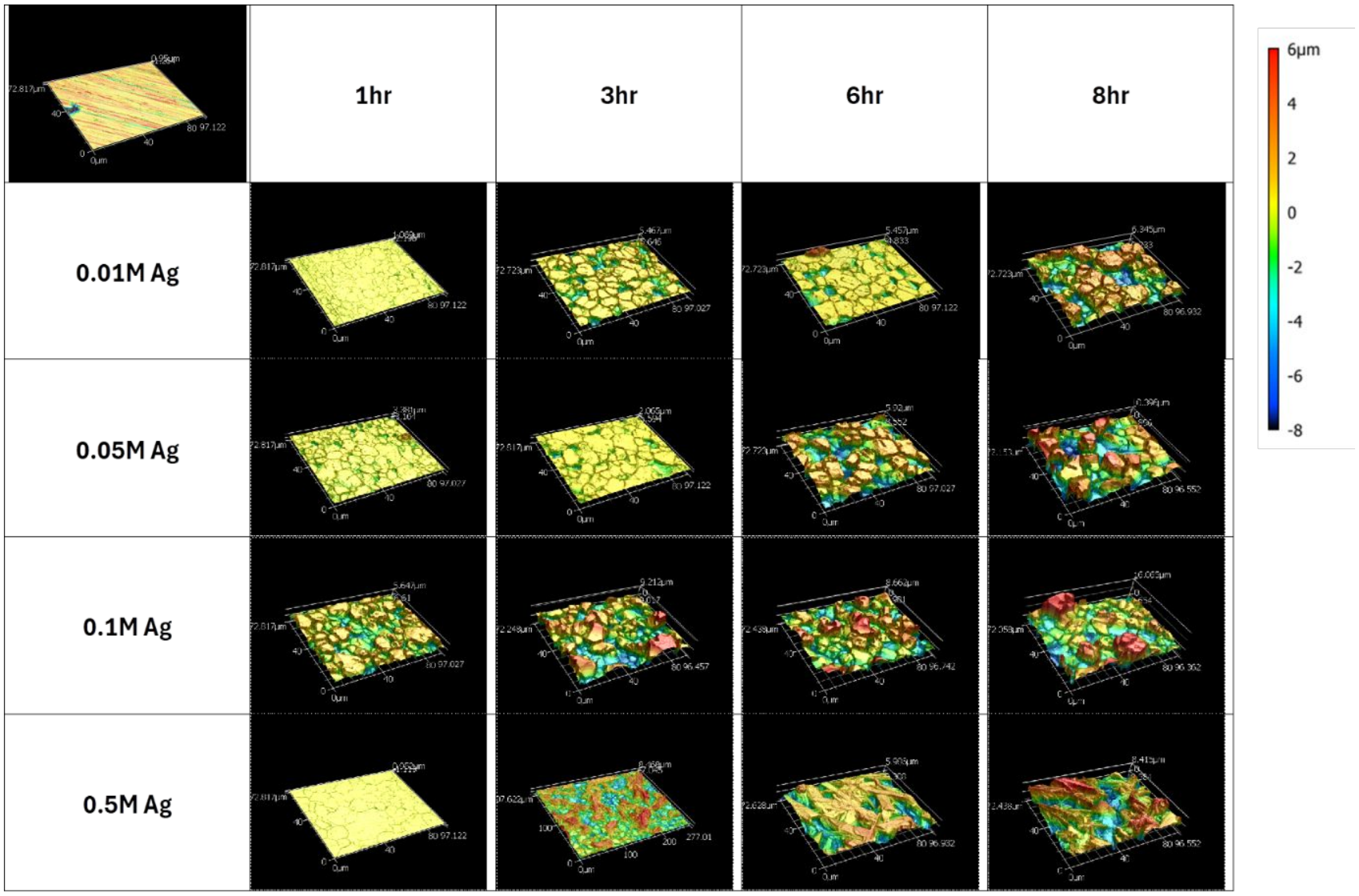

Figure S27. Surface profiler images of SS316 surfaces at 25× magnification. Pristine SS316 is shown at the top corner. Control samples were exposed to 8M HNO_3. Samples of SS316 were exposed to Ag(II) over time. Conditions: initial $AgNO_3$ stock = variable $AgNO_3$ concentration (0.01, 0.05, 0.1, and 0.5M) in 8 M nitric acid, catholyte solution = 8 M nitric acid, temperature = 25 °C.

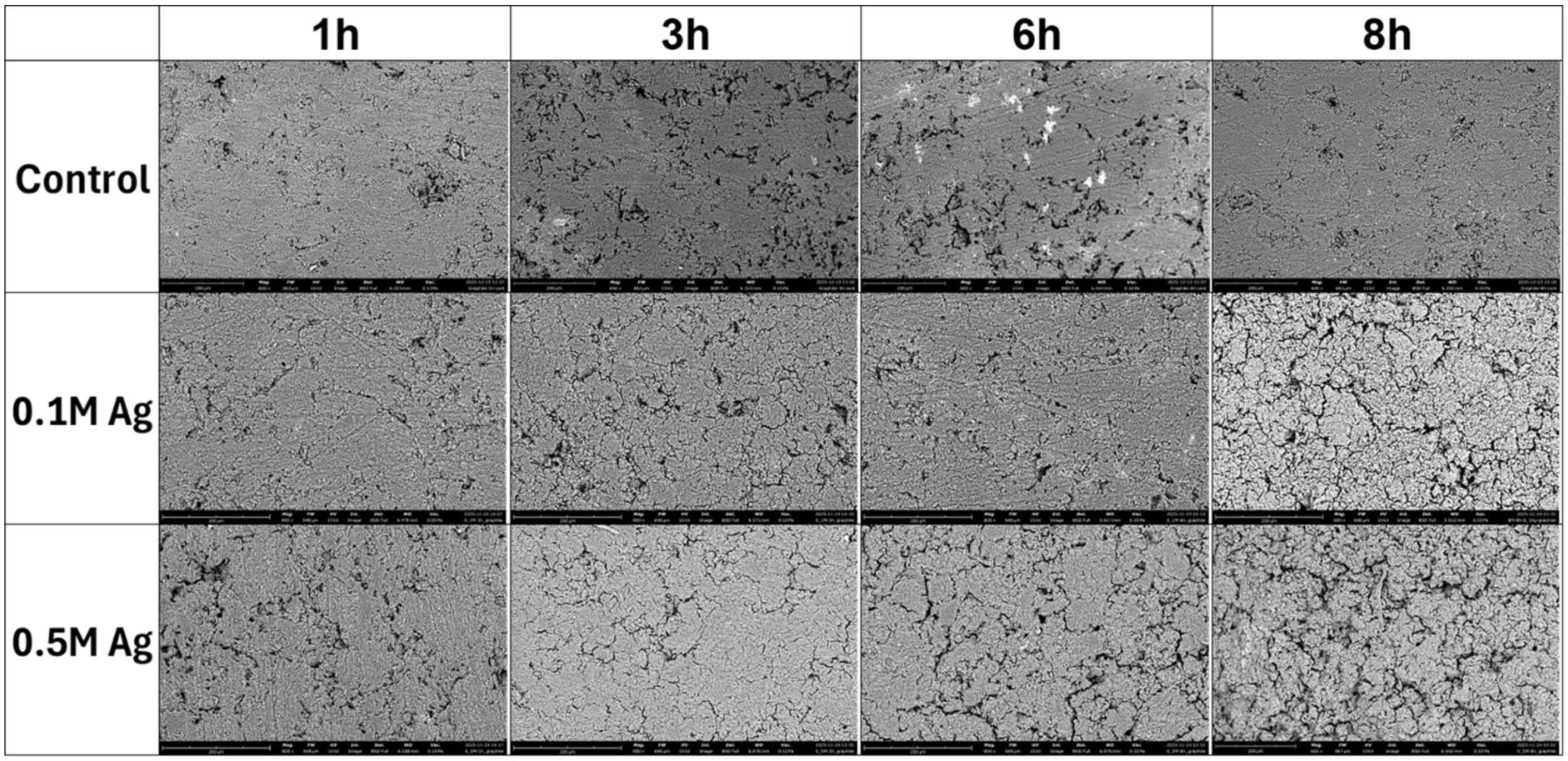

Figure S28. Surface profiler images of graphite surfaces at 25× magnification. Control samples were exposed to 8M HNO_3. Samples of graphite were exposed to Ag(II) over time. Conditions: initial $AgNO_3$ stock = variable $AgNO_3$ concentration (0.1 and 0.5M) in 8 M nitric acid, catholyte solution = 8 M nitric acid, temperature = 25 °C.

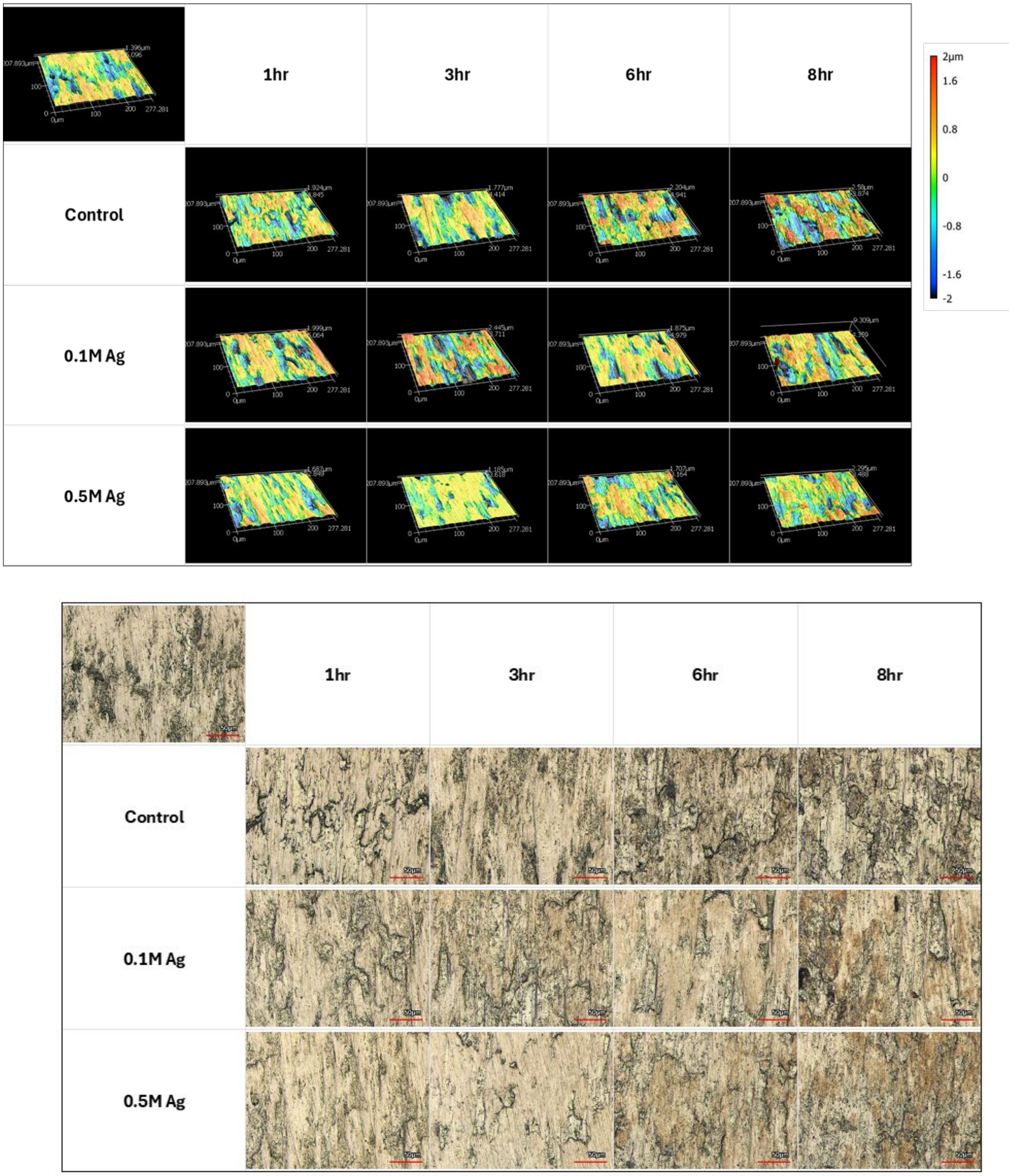

Figure S29. Surface profiler images of titanium surfaces at 25× magnification. Pristine titanium is shown at the top corner. Control samples were exposed to 8M HNO_3. Samples of titanium were exposed to Ag(II) over time. Conditions: initial $AgNO_3$ stock = variable $AgNO_3$ concentration (0.1 and 0.5M) in 8 M nitric acid, catholyte solution = 8 M nitric acid, temperature = 25 °C.

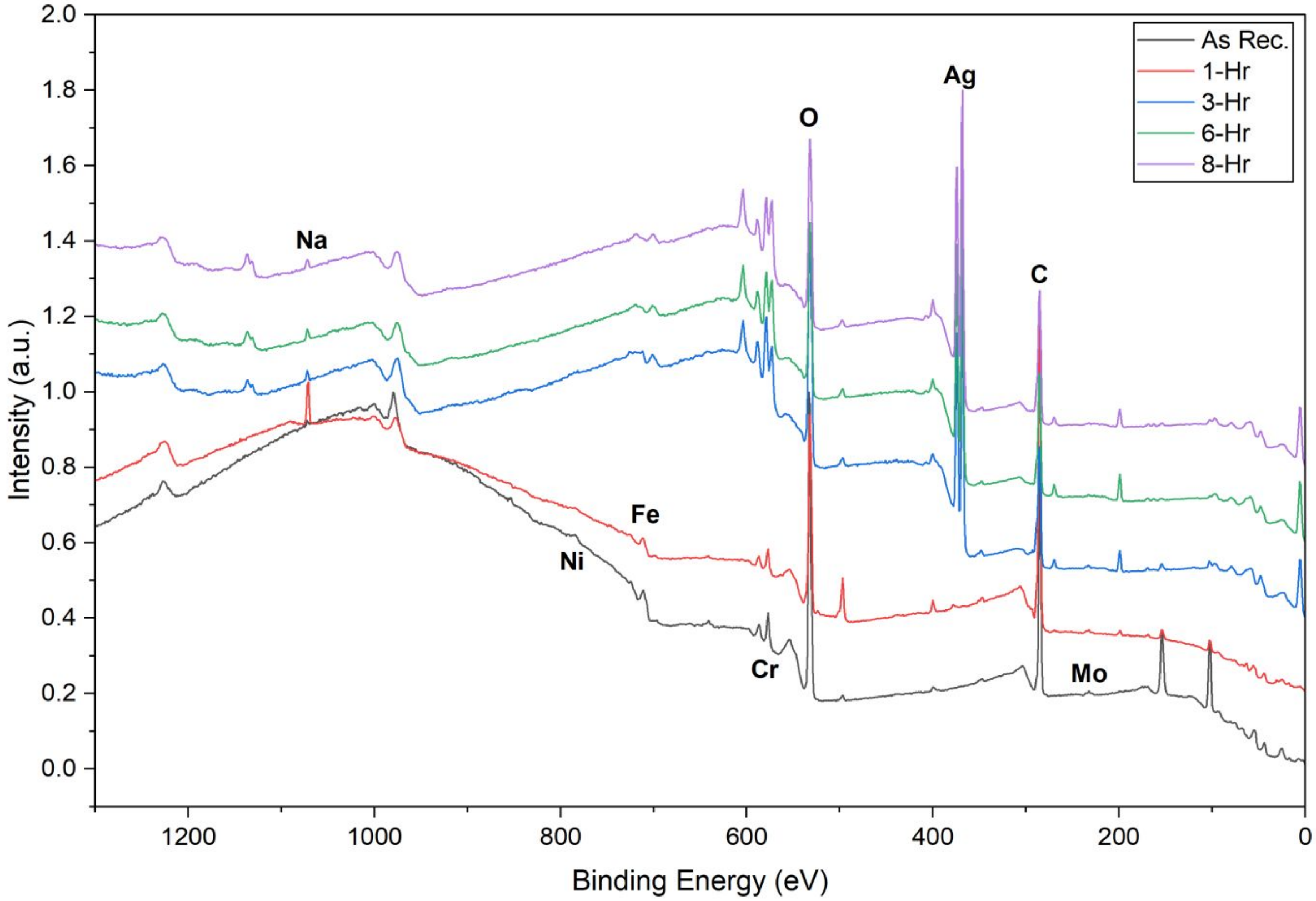

Figure S30. XPS survey spectra of SS316 samples before and after reacted with Ag(II). Samples were pulled at 1, 3, 6, and 8 hours. Conditions: initial $AgNO_3$ stock = 0.5M $AgNO_3$ concentration in 8M HNO_3, catholyte solution = 8M HNO_3. The spectra show the presence of Fe, Cr, Ni, and Mo from the stainless-steel substrate, along with O and C from surface oxides and adventitious carbon. Minor elements (Na, Si, Cl, Ca, and K) are attributed to environmental contamination. Silver is observed only in the exposed samples, with increasing intensity as a function of exposure time, indicating progressive surface deposition.

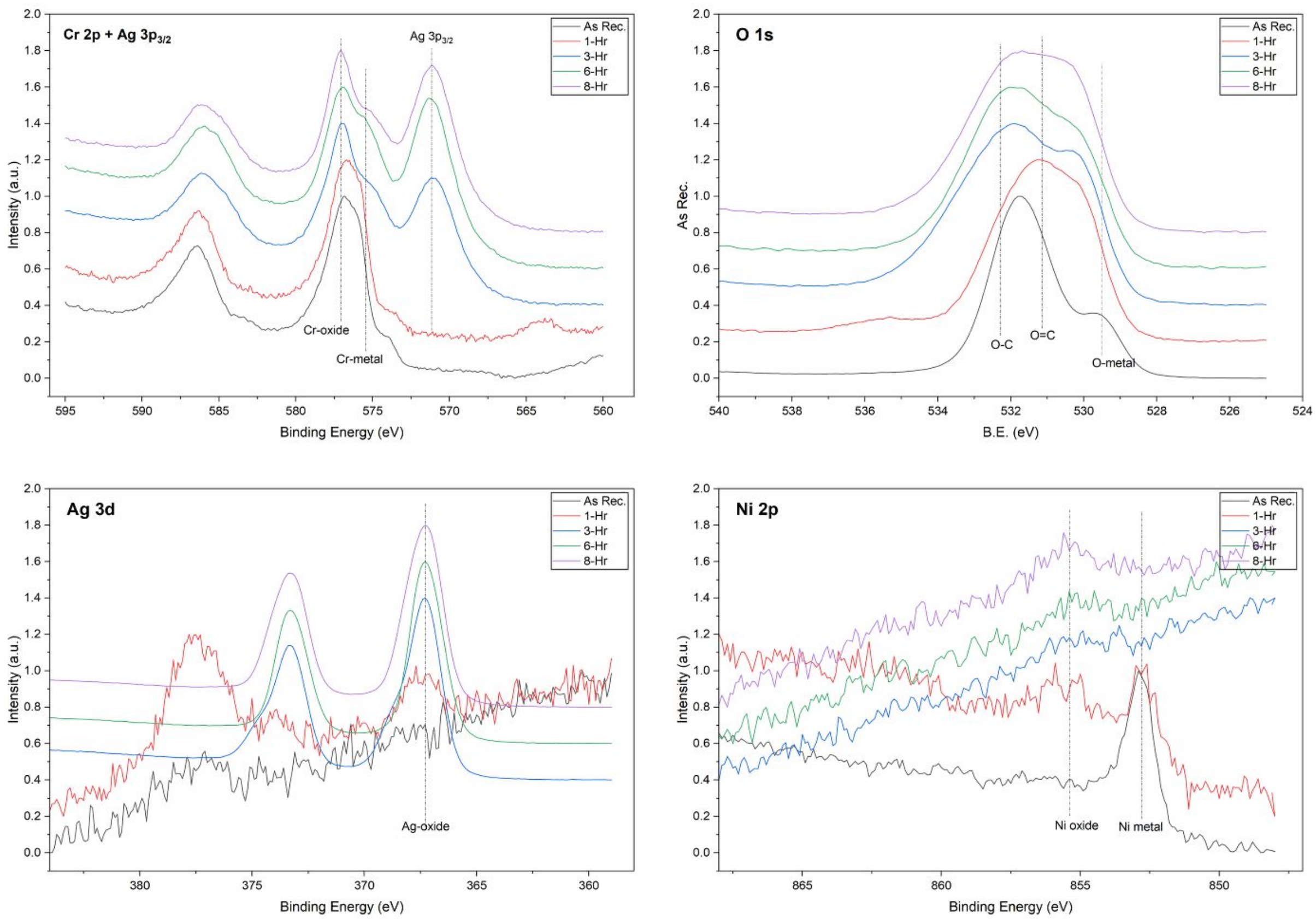

Figure S31. XPS spectra of (Top left) Cr 2p/Ag 3p, (top right) O 1s, (bottom left) Ag 3d, and (bottom right) Ni 2p regions for SS316 samples before and after reacted with Ag(II). Samples were pulled at 1, 3, 6, and 8 hours. Conditions: initial $AgNO_3$ stock = 0.5M $AgNO_3$ concentration in 8M HNO_3, catholyte solution = 8M HNO_3.

Uncertainty and Assumptions in GIXRD Depth Analysis

The penetration depths estimated from GIXRD measurements rely on several simplifying assumptions. Calculations assume a laterally uniform $Ag_2Cr_2O_7$ surface layer with homogeneous composition, constant density and negligible surface roughness. In practice, microscopy indicates that the reaction layer is neither perfectly dense nor laterally uniform and may exhibit porosity, thickness variations and preferred orientation. As a result, the calculated penetration depths should be regarded as effective or upper-bound estimates rather than precise physical thickness. Additionally, the use of bulk mass absorption coefficients and idealized beam geometry neglects potential contributions from surface roughness, grain statistics and microstructural shadowing effects, which may influence relative peak intensities at low ω angles. Despite these uncertainties, the qualitative trends in substrate peak emergence with increasing ω angles provide a robust indicator of the approximate depth scale over which the surface reaction layer attenuates the incident beam (the 0.8°–12° scans mentioned earlier).
